# Berry Anthocyanin, Acid, and Volatile Trait Analyses in a Grapevine-Interspecific F2 Population Using an Integrated GBS and rhAmpSeq Genetic Map

**DOI:** 10.3390/plants11050696

**Published:** 2022-03-04

**Authors:** Dilmini Alahakoon, Anne Fennell, Zachary Helget, Terry Bates, Avinash Karn, David Manns, Anna Katharine Mansfield, Bruce I. Reisch, Gavin Sacks, Qi Sun, Cheng Zou, Lance Cadle-Davidson, Jason P. Londo

**Affiliations:** 1 Department of Agronomy, Horticulture and Plant Science, South Dakota State University, Brookings, SD 57007, USA; dilmini.alahakoon@sdstate.edu (D.A.); zachary.helget@jacks.sdstate.edu (Z.H.); 2 Department of Food Science, Cornell University, Ithaca, NY 14853, USA; tlb247@cornell.edu (T.B.); gls9@cornell.edu (G.S.); 3 School of Integrative Plant Science, Cornell AgriTech, Cornell University, Geneva, NY 14456, USA; avi.karn6@gmail.com (A.K.); bruce.reisch@cornell.edu (B.I.R.); jpl275@cornell.edu (J.P.L.); 4 Department of Food Science, Cornell AgriTech, Cornell University, Geneva, NY 14456, USA; dcm38@cornell.edu (D.M.); akm87@cornell.edu (A.K.M.); 5 Computational Biology Service Unit, Life Sciences Core Laboratories Center, Cornell University, Ithaca, NY 14853, USA; qisun@cornell.edu (Q.S.); cz355@cornell.edu (C.Z.); 6 USDA-ARS, Grape Genetics Research Unit, Geneva, NY 14456, USA; lance.cadledavidson@usda.gov

**Keywords:** anthocyanin diglucoside, berry volatile, (E)-2-hexenal, malic acid, grapevine, *Vitis riparia*, ‘Seyval blanc’, QTL

## Abstract

Increased map density and transferability of markers are essential for the genetic analysis of fruit quality and stress tolerance in interspecific grapevine populations. We used 1449 GBS and 2000 rhAmpSeq markers to develop a dense map for an interspecific F_2_ population (VRS-F_2_) that was derived by selfing a single F_1_ from a *Vitis riparia* x ‘Seyval blanc’ cross. The resultant map contained 2519 markers spanning 1131.3 cM and was highly collinear with the *Vitis vinifera* ‘PN40024’ genome. Quantitative trait loci (QTL) for berry skin color and flower type were used to validate the map. Four rhAmpSeq transferable markers were identified that can be used in pairs (one pistillate and one hermaphroditic) to predict pistillate and hermaphrodite flower type with ≥99.7% accuracy. Total and individual anthocyanin diglucoside QTL mapped to chromosome 9 near a *5-O-GLUCOSYLTRANSFERASE* candidate gene. Malic acid QTL were observed on chromosome 1 and 6 with two *MALATE DEHYRDROGENASE CYTOPLASMIC 1* and *ALUMINUM-ACTIVATED MALATE TRANSPORTER 2-LIKE* (*ALMT*) candidate genes, respectively. Modeling malic acid identified a potential QTL on chromosome 8 with peak position in proximity of another ALMT. A first-ever reported QTL for the grassy smelling volatile (E)-2-hexenal was found on chromosome 2 with a *PHOSPHOLIPID HYDROPEROXIDE GLUTATHIONE PEROXIDASE* candidate gene near peak markers.

## 1. Introduction

Grapevine [*Vitis* sp.] is a perennial woody fruit crop species with high economic and nutritional value [1]. Typical grapevine primary breeding objectives include greater yield and higher quality, tolerance to biotic and abiotic stresses, and desirable plant growth habits [1]. However, the heterozygosity and long generation cycle of *Vitis* present a breeding challenge [1,2,3]. Molecular mapping with transferable markers provides the opportunity to reduce the time needed to study interspecific populations by facilitating quantitative trait loci (QTL) identification, marker assisted selection, and candidate gene discovery [1,3,4]. The results of QTL mapping vary among populations (size and genetic background) and marker type; therefore, the development of a genetic map with strong marker transferability is a timely requirement for grapevine breeding [4].

Restriction enzyme-based GBS methods strongly influenced genetic map construction over the past ten years and have several advantages such as, the possibility to apply to any species without prior genomic knowledge, simultaneous marker discovery and genotyping, low cost, high throughput, and scalability [5,6,7]. Limitations are that GBS marker development requires high-quality DNA to prevent heterozygous genotypes being wrongly called as homozygous (heterozygous under calling), generally targets gene-rich regions, and interspecific marker transferability can be as low as 2% [5,6,8]. In contrast, development of rhAmpSeq core genome markers needs diverse species genome sequences to target collinear regions with moderate polymorphism and produces fewer markers than GBS technology; however, these local haplotype markers are more informative and have high transferability across the *Vitis* genus [8].

Grapevine breeding and trait mapping have proceeded rapidly with the availability of genome sequences, molecular marker implementation and evolution, and increased use of interspecific crosses for the introgression of economic traits [6]. Berry skin color, a qualitative trait, has been mapped to a major locus on chromosome 2 and associated with *MYBA1* gene [4,9,10,11,12,13,14]. Anthocyanins (concentration and type) influence berry color and contribute to wine quality and have been mapped to chromosome 2, 7, 12, 13, and 14 [15,16,17]. Another qualitative trait, flower type, also maps to chromosome 2 [8,18,19,20,21,22,23,24]. Thus, berry color and flower type provide well studied reference points for validating and comparing genetic maps and genome-wide association studies in populations generated from *V. vinifera* L., *V. rotundifolia* Michx., and interspecific crosses [4,10,25].

Quantitative traits that are of high value for molecular assisted selection (disease resistance, agronomic traits, and berry chemistry) are rapidly being assessed to identify their relevant loci and candidate genes [11]. With many breeding programs focused on increased disease resistance, the introgression of non-vinifera species has also increased the emphasis on berry chemistry traits as non-vinifera species can contribute different fruit characters that may impact product quality. QTL analyses for standard fruit harvest parameters (berry acids, soluble solids, and pH) show multiple loci varying with population and year [26,27,28,29,30]. Fewer volatile QTL have been identified in berries, although many genes and enzymes associated with volatile development in berries have been detected [31]. The methoxypyrazines (3-isobutyl-2-methoxypyrazine (IBMP), isopropyl methoxypyrazine (IPMP)) and C_6_ volatiles (hexanal, hexenal) are of particular interest as these herbaceous volatiles are found at high concentrations in wild *Vitis* spp. and can contribute positive and negative odors depending on their concentrations [32,33,34,35]. For example, IBMP contributes vegetable-like aromas that may be a positive attribute in some white wines (i.e., ‘Sauvignon blanc’) but are typically considered negative in red wines (i.e., ‘Cabernet Sauvignon’, ‘Cabernet Franc’, and ‘Merlot’) [36]. Five QTL explaining 40% of the grapevine leaf IBMP are found in a *V. vinifera* ‘Cabernet Sauvignon’ x *V. riparia* ‘Riparia Gloire Montpelier’ population [37]. A F_2_ population derived from Cabernet Sauvignon and Pinot noir shows a berry IBMP locus and candidate genes located on chromosome 3 [38]. In contrast, no QTL are reported for the herbaceous C_6_ aldehyde volatiles, which are products of the fatty acid break-down through the lipoxygenase pathway, although several enzymes have been identified for the development of C_6_ aldehyde and alcohol products [31].

The greater use of non-vinifera species in breeding programs provides the opportunity and challenge to identify the genetics of positive and negative fruit attributes to promote capture or removal of the traits through marker assisted selection. The objectives of this study were to: (1) construct a high-density linkage map using GBS and rhAmpSeq molecular markers for genetic studies in an interspecific F_2_ population, (2) validate this integrated map using the stable traits of flower type and color, and (3) determine berry anthocyanin, malic acid, titratable acidity (TA), and volatile QTL and associated candidate genes in an interspecific F_2_ population.

## 2. Results

### 2.1. Segregation Distortion Analysis and Distortion Threshold Estimation for VRS-F_2_ Population

**Figure 1:**
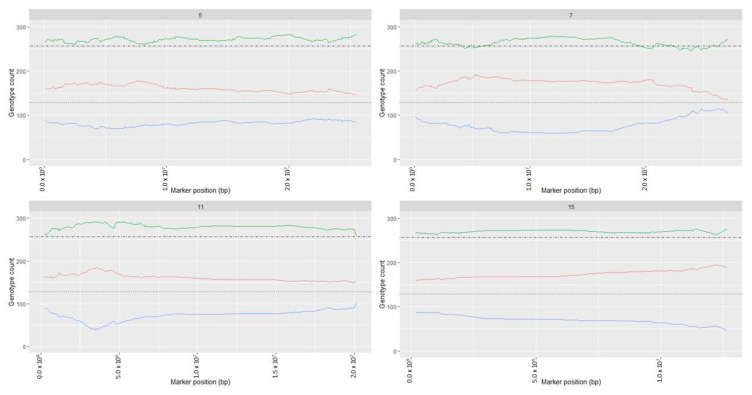
Genotype frequency plots for chromosomes 5, 7, 11, and 15. Horizontal lines represent expected genotype count 128 (for AA (red) and BB (blue), dotted) and 256 (for AB (green), dotdash) under 1:2:1 Mendelian ratio.

**Figure 2:**
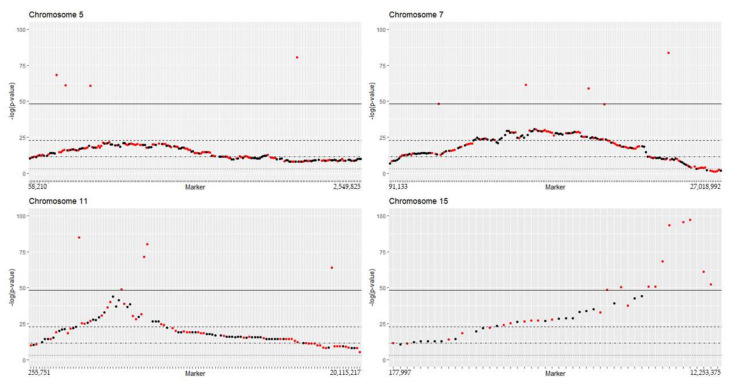
Marker segregation distortion at four separate chi-square adjusted *p*-value threshold levels for chromosome 5, 7, 11, and 15. The lines represent negative log scale adjusted *p*-value < 5 × 10^−2^ (dot), 1 × 10^−5^ (dot-dash), 1 × 10^−10^ (dash), and 1 × 10^−21^ (solid). The rhAmpSeq (red) and GBS (black) markers show distorted marker type at each threshold level. Markers above −log(1 × 10^−100^) are not in the figures.

**Figure 3:**
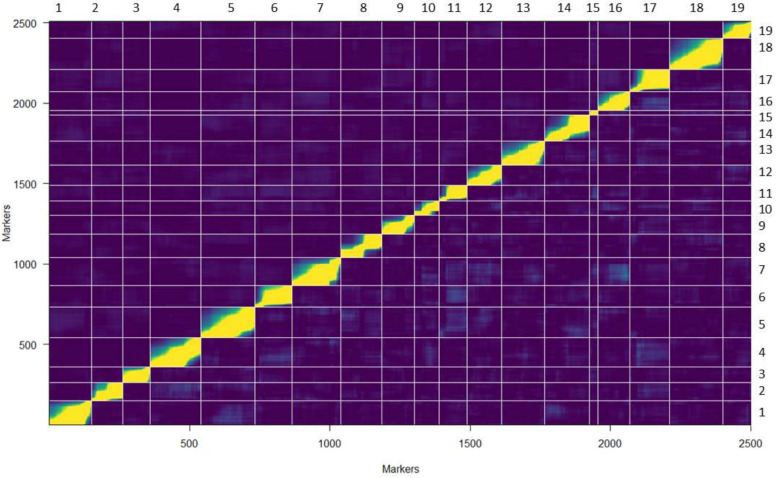
Pair-wise recombination fractions and LOD of the VRS-F_2_ genetic map. Vertical and horizontal lines indicate the borders of the linkage groups. The estimated recombination fractions (*r*) between markers are in the upper left and the LOD are in the lower right of each linkage group rectangle. High correlation between markers indicates marker linkage (yellow, low $\hat{r}$ or high LOD) and blue (high $\hat{r}$ or low LOD) represents low correlation values indicating unlinked markers.

Genotype frequency plots for markers in the linkage map showed deviation from an expected 1:2:1 ratio for many markers associated with chromosomes 5, 7, 11, and 15 (Figure 1 and Figure S1). The AA genotype was more frequent and BB genotype less frequent than the expected 1:2:1 Mendelian ratio (Figure 1). While segregation distortion typically was in the middle of the chromosome, distortion for chromosome 15 was at the end (Figure 2, Table S1). This confirmed the presence of some chromosomal regions with natural segregation distortion patterns that needed to be preserved in the map. Analysis of a series of *p*-values indicated >1210 markers would be removed at the adjusted *p*-value 0.05 (Figure S2). To conserve markers representative of the natural genetic character of the VRS-F_2_ population, the threshold (adjusted *p*-value) was set at <10–21 for this F_2_ population and pairwise marker linkage analysis was conducted. No marker ordering errors were identified in this final map (Figure 3). A total of 677 non-informative markers and 232 distorted markers were identified and removed during map curation and the final map contained 2519 markers (Table 1).

The overall genome coverage of mapped markers (96.3%) and collinearity (99.9%) relative to the *V. vinifera* PN40024 12X V2 genome indicated high genetic map quality (Table 1 and Table 2, Figure S3). All chromosomes had >93% genome coverage except for chromosome 15 with 61.5% coverage (Table S1). Eighty-nine markers were tested for chromosome 15, of which 41 were non-informative and 14 were distorted resulting in only 34 markers for this chromosome. The resultant VRS-F_2_ GBS-rhAmpSeq integrated map had a total length of 1131.3 cM and an average distance of 0.5 cM between markers (Table 1 and Figure S4).

### 2.2. Map Validation with Berry Skin Color and Flower Type

Two well-studied binary traits were used to validate the VRS-F_2_ GBS-rhAmpSeq map: grapevine berry skin color and flower type (pistillate, hermaphroditic). There were 22 white and 78 black fruited genotypes observed in the fruiting field vines. Chi-squared test did not reject the hypothesis of a 3:1 ratio of black:white (chi-squared value = 0.56 and *p*-value = 0.46) consistent with a single locus and black being dominant over white skin color. Berry skin color mapping identified a significant QTL on chromosome 2 with 22.8 LOD value and peak position at 13.5 Mb position in the map (Table 3). The QTL confidence interval contained many MYB family genes including the MYBA1 gene associated with berry color. Flower type was mapped to chromosome 2 using 97 genotypes. The VRS-F_2_ parent (16_9_2) was heterozygous (Hf) for the dominant hermaphroditic flower type and analysis of the F_2_ progeny field vines flower type indicated 81:16 hermaphroditic (HH or Hf): pistillate (ff) supporting the expected 3:1 ratio (chi-squared value = 2.61 and *p*-value = 0.11). A flower type QTL was detected on chromosome 2 at 4.65 (Mb) with 17.2 LOD and a very narrow 95% Bayesian interval (Table 3). Seven markers including markers within the QTL confidence interval (rh_2_4497054, GBS_2_4567885, rh_2_4599939, GBS_2_4650201), two markers nearby the confidence interval (rh_2_4703733 and GBS_2_5352479), and a previously reported marker (rh_2_4825658) were used to predict flower type phenotype based on genotype. These markers predicted that the 358 unknown individuals were potentially 1:1:2 homozygous pistillate, homozygous hermaphroditic, and heterozygous hermaphroditic, respectively (Table S2). The training set of 97 individuals showed varying marker accuracy depending on flower type. Four markers showed 100% prediction accuracy for the pistillate phenotype and prediction for hermaphroditic types ranged from 96 to 99% accuracy across the seven markers (Figure S5). A test data set of 59 newly identified flower type phenotypes gathered in greenhouse-grown vines was used to validate the accuracy of genotype prediction and a 100% accuracy was obtained for the pistillate phenotype in comparison to the genotype prediction for all seven markers. The accuracy for the hermaphroditic phenotype was similar with 95 to 98% accuracy depending on the marker. Transferable marker pairs, one pistillate (rh_2_4497054 or rh_2_4599939) and one hermaphroditic (rh_2_4703733 or rh_2_4825658) could be used for pistillate (ff) and hermaphroditic (HH or Hf) identification with 100 and 99.7% overall accuracy, respectively.

### 2.3. Berry Anthocyanin, Acid, and Volatile Analyses

**Figure 4:**
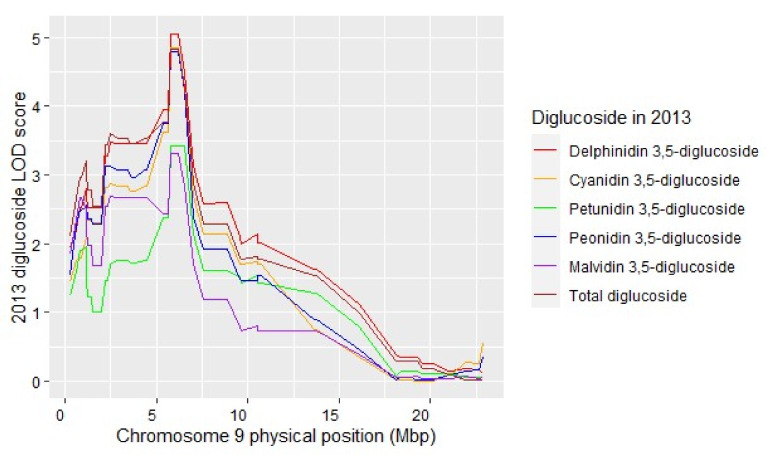
Individual anthocyanin diglucosides and total diglucoside LOD scores for 2013.

The total anthocyanins and total mono- and diglucoside anthocyanins concentrations in the F_2_ progeny ranged from less than that of 16_9_2 (parent of VRS-F_2_ population) to a concentration about as great as that of the *V. riparia* female grandparent (Table S3). Total anthocyanin QTL peak positions on chromosome 2 were close to the berry skin color QTL peak position (Table 3). In contrast, the total mono- and diglucosides QTL peak positions were upstream of the total anthocyanin peak position and only malvidin and petunidin 3-glucoside QTL occurred on chromosome 2 in two years (Table S4). Delphinidin, malvidin and petunidin 3,5-diglucosides QTL were present on chromosome 2 in 2013 only. However, the cyanidin, malvidin, peonidin, and petunidin 3,5 diglucoside QTL were collocated on chromosome 9 in both years (Figure 4 and Table S4). The peak marker for the diglucosides is near the *5-O-GLUCOSYLTRANSFERASE* (Vitvi09g00582) candidate gene at 6.52 Mb in *V. vinifera* PN40024 12XV2. *V. riparia* ‘Manitoba 37’ is homozygous for increased diglucoside concentration and ‘Seyval blanc’, which contains *V. rupestris* Scheele in its pedigree, is heterozygous for the marker rh_9__6523189 at the 5*-O-GLUCOSYLTRANSFERASE* gene. Protein alignment of Vitvi09g00582 with the corresponding *V. riparia* Michx (Accession *#*XP_034695482.1), *V. amurensis* Rupr. (Accession #AHL68667.1), and *V. rotundifolia* (Accession *#*ALS55360.1) indicate that Vitvi09g000582 has an early truncation; whereas, the three native species contain an additional segment with 51 amino acids (Figure S6).

**Figure 5:**
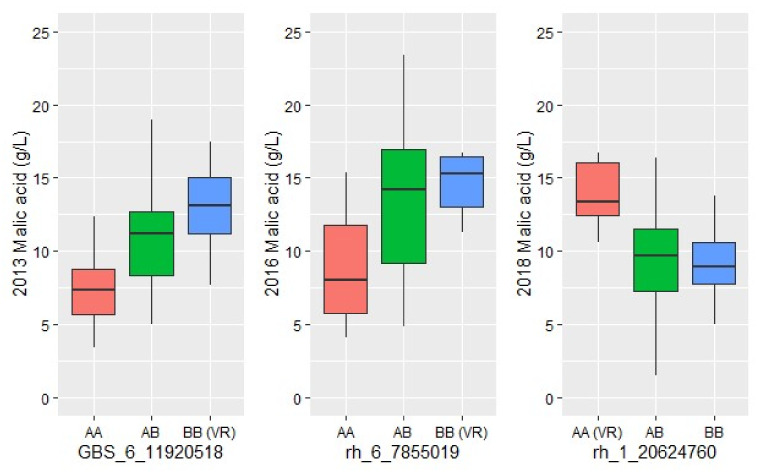
Effect plots for chromosome 6 (2013, 2016) and chromosome 1 (2018) malic acid QTL peak position markers. x-axis is the genotype for each marker and y axis is the malic acid concentration. The *V. riparia* ‘Manitoba 37’ (pistillate grandparent) genotype contributing to high malic acid is indicated in parenthesis on x-axis.

Malic acid concentration ranged from 1.5 to 29.0 g/L across years and generations (Table S3). In the F_2_ population, mean malic acid content ranged from 10.6 to 12.0 g/L across years. Correlation analysis showed strong, positive, and significant pairwise correlations for malic acid for most years (Table S5). QTL for malic acid were detected on chromosome 6 in all years and on chromosome 1 for 2016 and 2018 (Table 3 and Table S5). Collocated potential malic acid QTL (overlapping confidence intervals) were observed on chromosome 8 in 2016 and 2018 and these were significant in the malic acid model (Figure S7, Table S6). QTL modeling in 2016 indicated a significant interaction was observed between QTL on chromosome 1 and 8 (Table S6). Additive modeling of QTL on chromosomes 1, 6, and 8 explained more than 50% of the malic acid variation. Collocation of the TA and malic acid QTL was observed on chromosome 6 at 7.86 Mb in 2016 and on chromosome 1 at 5.59 Mb in 2018. Mean trait effect plots by genotypes for malic acid peak positions on chromosome 1 and chromosome 6 indicated *V. riparia* ‘Manitoba 37’ was the responsible grandparent for high malic acid in all years (Figure 5). Two *MALATE DEHYDROGENASE* (Vitvi01g002239 and Vitvi01g002240) and one *MALATE DEHYDROGENASE PRECURSOR* (Vitvi01g01744) were identified as candidate genes within the 95% confidence interval for the chromosome 1 malic acid QTL. Two *ALUMINUM-ACTIVATED MALATE TRANSPORTER 2-LIKE* genes (Vitvi06g00922, Vitvi06g00928) were identified as candidate genes for malic acid QTL on chromosome 6. In addition, modeling of malic acid identified a potential QTL on chromosome 8 in 2016 and 2018. An *ALUMINUM ACTIVATED MALATE TRANSPORT 8-LIKE* (Vitvi08g00636) was also identified in the confidence interval of that QTL. The Vitvi08g00636 and Vitivi08g00142 genes are closest to QTL peak positions.

The mean TA ranged from 12.88 to 28.28 g/L across three years (2013, 2016, and 2018) and generations (grandparents, parent, and F_2_ population mean) (Table S3). The VRS-F_2_ TA had strong positive correlations across three years (0.82 to 0.89) and there was a strong correlation with malic acid (Table S5). Five significant QTL were observed for TA, on chromosome 6 for all three years and on chromosome 1 in 2016 and 2018 (Table 3). Temperature data (June through August) shows that 2013 was cooler in early season than 2016 and 2018 (Table S7).

**Figure 6:**
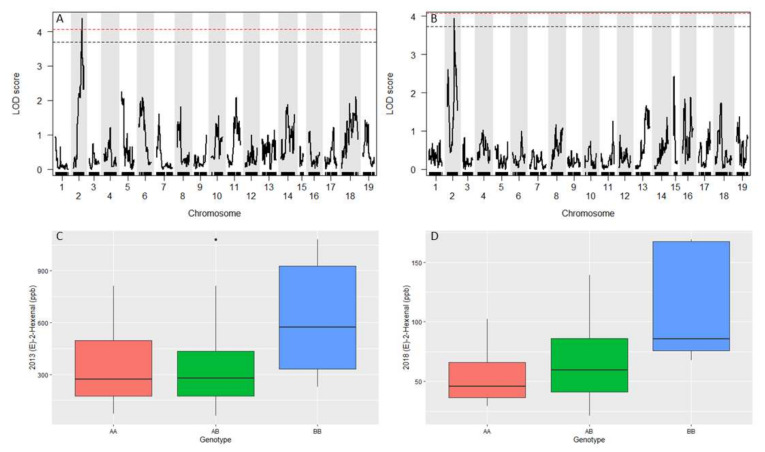
Genome-wide LOD for (E)-2-hexenal in 2013 (**A**) and 2018 (**B**). Black and red dashed line represent 1000 permutation at alpha of 0.1 and 0.05, respectively. (E)-2-hexenal averaged across 13 markers between peak positions for 2013 (**C**) and 2018 (**D**). *V. riparia* ‘Manitoba 37’ grandparent (pistillate) is AA for all markers. ‘Seyval blanc’ (staminate) is BB for 11 markers and AB for 2 of the markers.

Berry volatiles varied by year and no hexanal, IBMP or IPMP QTL were identified although there was variation in concentration of these volatiles (Table S3). A QTL was identified for berry volatile compound (E)-2-hexenal on chromosome 2 in 2013 and 2018 (Table 3, Figure 6A,B). Analysis of 13 markers between peak positions of 2013 and 2018 QTL indicated that the ‘Seyval blanc’ grandparent contributes to the greater (E)-2-hexenal concentration in this population (Figure 6C,D). The 95% confidence intervals for the (E)-2-hexenal QTL were large and contained many fatty acid metabolism genes. It is noteworthy however, that a PHOSPHOLIPID HYDROPEROXIDE GLUTATHIONE PEROXIDASE gene (Vitvi02g00663) was found near the peak markers for 2016 and 2018. Two additional *PHOSPHOLIPID HYDROPEROXIDE GLUTATHIONE PEROXIDASE* genes were found within the 2018 confidence interval.

## 3. Discussion

### 3.1. Natural Segregation Distortion Was Significant in F_2_ Population

The current VRS-F_2_ GBS-rhAmpSeq integrated map includes markers that deviate from Mendelian segregation 1:2:1 ratio for chromosome 5, 7, 11, and 15. The addition of some of the distorted markers did not show any effect on marker order or recombination percentage, suggesting that marker segregation distortion is natural in these chromosomal regions for this F_2_ grapevine population [39,40]. The inclusion of these markers increased marker density and filled long gaps in the genetic map on chromosomes 5, 7, 11, and 15. Within the distorted regions, several reproductive related genes (multiple reproductive (Vitvi05g00109, Vitvi07g00910, Vitvi07g00921), male-sterility (Vitvi07g00807, Vitvi07g00814, Vitvi07g00822) and embryo development (Vitvi05g01066, Vitvi07g01429, Vitvi15g00642, Vitvi15g01065, Vitvi15g01084, Vitvi15g01085, Vitvi15g01122) were identified. Selection pressure operating against one of the parental alleles at meiosis cell division or zygote level has been suggested as a reason for natural segregation distortion in wheat [40]; however, it is not possible to determine whether this is a factor in this study. Segregation distortion can also be related to non-biological reasons, such as a small sampling population or genotype errors [41]. However, the mapping population size (514 individuals), advanced marker techniques, and the continuous blocks of segregation distortion suggest that biological, not technical, factors caused the segregation distortion in the VRS-F_2_ population. Awareness of the distorted regions may help in planning crosses; however, further investigations are necessary to identify the mechanism(s) causing the segregation distortion.

### 3.2. The VRS-F_2_ GBS-rhAmpSeq Integrated Map Provides Greater Marker Transferability to Other Populations

Genetic maps play an important role in the identification of QTL, candidate genes, and marker assisted selection. Despite technological advances, low marker density and quality, high cost, and low marker transferability across populations remain as challenges in genetic map construction. Previous genetic maps for this VRS-F_2_ grapevine population developed using SSR and GBS markers had large gaps in coverage [42,43]. In this study rhAmpSeq markers, which have the potential of high marker transferability to other *Vitis* germplasm, were used in combination with GBS markers to construct a high-density integrated genetic map for the interspecific F_2_ population. The current integrated genetic map showed improvements over the previous GBS map for this population, with an increased marker density (74%) and increased genomic coverage. In addition, the inflated length of the previous GBS genetic map was reduced from 2424 to 1131 cM. Correlation with the *V. vinifera* PN40024 12X V2 genome was higher than 98% for 18 of the 19 chromosomes in the integrated map. Chromosome 15 had lower correlation coefficient and coverage; however, this VRS-F_2_ GBS-rhAmpSeq integrated map increased the chromosome 15 marker density (by 62% or 13 markers) relative to the previous GBS map [43]. The integrated map incorporates 1070 rhAmpSeq markers that were developed using multiple *Vitis* species [8]. The rhAmpSeq markers identified for traits in this population can thus be used for marker-assisted selection if the GBS markers fail in other interspecific populations.

### 3.3. Confirmation of Berry Skin Color and Flower Type Loci

Berry color is produced by synthesis and accumulation of anthocyanins in the berry skin and MYB and bHLH family genes have been identified as contributing to skin color development [17,44,45]. The VvMYBA1 gene on chromosome 2 has been identified as one of the main genes for berry color [9]. In the integrated VRS-F_2_ GBS-rhAmpSeq integrated map, the QTL peak position 14.11 Mb is close to the VvMYBA1 gene. Several studies, in different grapevine genetic backgrounds, place the flower type locus on chromosome 2 [21,22,46]. Recently, a 150 kb flower type determination region was described on chromosome 2 that contains a proposed pistillate sterility locus (VviYABBY) and staminate sterility locus (VviINP) [24,47]. A QTL for pistillate flower type in this VRS-F_2_ population at 4.65 Mb is close to a rhAmpSeq marker (rh_2_4825658) identified by GWAS analysis of interspecific crosses [6]. It is also upstream of the C region of the proposed flower type determining region containing the putative staminate sterility gene VviINP [24,47]. A survey of the markers on either side of QTL peak marker GBS_2_4650201 indicated the three upstream markers (rh_2_4497054, GBS_2_4567885, rh_2_4599939) predicted pistillate flower type more accurately than those downstream. Thus, in addition to two GBS markers, there are four rhAmpSeq markers, two for pistillate (rh_2_4497054 and rh_2_4599939), and two for hermaphroditic flower types (rh_2_4703733 and rh_2_4825658) that can be used in combination to accurately predict these flower types. A genome-wide association study identified one transferable marker [6].

### 3.4. Individual Diglucoside Anthocyanin QTL Colocate

Anthocyanin pigment content and composition are critical to the color of berries and resultant wines. The major anthocyanidins (i.e., the aglycone component) found in grapes are malvidin, cyanidin, peonidin, petunidin, and delphinidin, which may exist in both mono- and diglucoside forms [48]. Anthocyanin diglucosides are at negligible concentrations in *V. vinifera*, but they are commonly found in wild *Vitis* spp. and their hybrids [48]. Diglucosides are generally considered undesirable for wine grapes because they are less able to form stable polymeric pigment; therefore, their presence is used as a bio-marker to identify unallowable interspecific hybrids in certain wine regions [16,49]. Costantini et al. 2015 [50] reported several minor QTL on multiple chromosomes which are not observed in this study. Individual and total diglucoside QTL were detected with a peak position near the *V. vinifera 5-O-GLUCOSYLTRANSFERASE* (Vitvi09g000582) candidate gene at 6.52 Mb. This gene, described by Jánváry et al 2009 [51], lacks 51 amino acids due to a premature stop codon and is non-functional in *V. vinifera*, resulting in the absence of anthocyanin 3,5-diglucosides in most *V. vinifera*. The 3,5- diglucoside anthocyanins are found in many of the other *Vitis* species including *V. riparia*. Protein alignment of Vitvi09g000582 with the corresponding *V. riparia*, *V. amurensis* and *V. rotundifolia* sequences (Figure S6) indicates these native American species contain 51 amino acids in the region necessary for diglucoside development. Characterization of the *V. amurensis 5-O-GLUCOSYLTRANSFERASE* (Va5GT) *in vitro* shows that it can synthesize diglucosidic anthocyanins [52]. There were no genotypes with black berries without diglucosides; however, several black fruited VRS-F_2_ individuals were identified with very low concentration of diglucoside anthocyanins, providing opportunity for genotype selections for future crosses.

### 3.5. QTL Mapping Identified Malic Acid Dehydrogenase Candidate Gene on Chromosome 1 and Suggests Potential Temperature Influence

Most wild *Vitis* spp. are known to have higher TA and malic acid berry concentrations than *V. vinifera*, resulting in excessively sour wines and lower wine quality [53]. In this study, there was a significantly high correlation (ranging from 0.71 to 0.96, *p*-value < 0.0001) between the TA and malic acid concentration in all years, indicating that variation in the TA in this population can largely be explained by the variation in malic acid. Malic acid is mainly synthesized through Krebs cycle/sugar metabolism and degrades during ripening through the TCA cycle and respiration [54]. The activity of several enzymes critical to malate respiration, including malate dehydrogenase, are known to increase with higher temperature. In this study of 30-day post-veraison berries, a QTL on chromosome 1 in 2018 and 2016 (LOD > 3) contributed significantly to the malic acid models, although no QTL for chromosome 1 was detected in 2013. It is noted that 2013 had lower preveraison temperatures than did 2016 and 2018; however, mean malic acid concentrations were similar across all three years, likely because these differences in temperature occurred before respiration commenced. The chromosome 1 QTL in VRS-F_2_ contained three candidate genes; two *MALATE DEHYRDROGENASE CYTOPLASMIC 1* genes (Vitvi01g02239, Vitvi01g02240) are located near the QTL peak position on chromosome 1 and a third gene (*MALATE DEHYROGENASE PRECURSOR*, Vitvi01g01744) is within the Bayesian confidence interval.

The malic acid QTL on chromosome 6 was stable in the VRS-F_2_ population across the three years. The QTL confidence interval contains *ALMT* candidate genes (Vitvi06g00922, Vitvi06g00928), first identified with single year data in the GBS map [43]. The *ALMT* gene plays a role in malate and tartrate accumulation [43,55]. The *ALMT* genes are located with other genes that regulate cytoplasmic and apoplastic pH and it is noted that *ALMT* activity is limited by temperature and ripening processes [56]. The modeling of malic acid also detected a potential QTL on chromosome 8, the position of the peaks (10.3 Mb, 2016 and 11.0 Mb, 2018) was close to another *ALMT* gene. In addition, the peak positions of the potential chromosome 8 malic acid QTL in VRS-F_2_ are within the physical confidence interval reported for a malic acid QTL in a ‘Norton’ x ‘Cabernet Sauvignon’ population [30], thus providing support for the potential QTL identified here.

Several previous studies also report lack of consistent QTL for malic acid, TA, and numerous other grapevine berry quality traits [26,57,58,59]. In *V. vinifera* and *V. aestivalis* Michx. derived populations, malic acid QTL have been found on chromosome 6 and 8 in multiple years. However, several other malic acid QTL, with varying degrees of stability from year to year, have been identified in grapevine populations with at least one on every chromosome [28,29,30]. This study reports multiple QTL for malic acid and TA in the same growing season with additive QTL modeling of QTL on chromosome 1, 6 and 8 explaining >50% of the malic acid variation. The difference in QTL relative to season GDD supports the suggestion that different physiological mechanisms in different interspecific population may influence the number and stability of QTL found in interspecific populations [29].

### 3.6. Berry Volatiles and (E)-2-Hexenal QTL

The VRS-F_2_ population varied in volatile compound concentrations across generations and years tested. This study focused on methoxypyrazines (IBMP, IPMP) and C_6_ aldehyde volatiles, as previous work has shown that these herbaceous and undesirable odorant classes are at higher concentrations in wild *Vitis* spp. [33]. IBMP was not detected in berries in 2018 and was present in only 27% of genotypes in 2013. The mean IPMP concentrations in *V. riparia* ‘Manitoba 37’ pistillate grandparent and VRS-F_2_ were greater than those previously reported for *V. riparia*; however, several VRS-F_2_ genotypes had concentrations in the range reported for *V. riparia* [33]. In *V. vinifera*, methoxypyrazines decrease markedly (~90%) during berry maturation, and the high IPMP values observed in this study may also be because berries were sampled at 30 days post-veraison rather than at post-veraison intervals more typical of commercial wine production (~60 days) [60]. The IPMP content in the VRS-F_2_ population varied consistently in 2013 and 2018; however, no QTL was identified for this berry volatile.

The C_6_ aldehydes, hexanal and (E)-2-hexenal, were present in the post-veraison berries of all generations. A novel (E)-2-hexenal QTL located on chromosome 2 was observed in 2013 and 2018. The (E)-2-hexenal QTL were located adjacent to the upper end of the anthocyanin QTL confidence intervals. The (E)-2-hexenal volatile concentrations varied among years and were similar to those in post-veraison ‘Cabernet Sauvignon’ berries but higher than observed in Chinese wild grape cultivars and ‘Seyval blanc’ [32,61,62]. In ‘Cabernet Sauvignon’, both hexanal and (E)-2-hexenal were present from fruit set through ripening and concentrations peaked post-veraison with C_6_ alcohols peaking during late ripening [32]. Several enzymes and genes have been identified in the lipoxygenase pathway leading to the development of C_6_ aldehyde products after crushing [31,32]. In ‘Cabernet Sauvignon’, volatile concentrations were suggested to be controlled by a tight regulation of the alcohol dehydrogenase, alcohol acetyl transferase, and enal isomerase enzymes in the lipoxygenase pathway during berry development, with the C_6_ aldehyde products being most prevalent in the veraison and post-veraison and alcohols in the late ripening stage [32]. However, none of the genes identified previously for the lipoxygenase pathway were found on chromosome 2 [31]. The examination of the genes underlying the (E)-2-hexenal QTL in this study showed a *PHOSPHOLIPID HYDROPEROXIDE GLUTATHIONE PEROXIDASE* gene near the peak markers for 2016 and 2018. Phospholipid hydroperoxide glutathione peroxidases enzymes are reactive oxygen species (ROS) scavengers responsible for reducing hydroperoxides generated in the oxidation of fatty acids by lipoxygenase hydroperoxide lyase [63]. It is noted that ROS scavenging enzymes increase at veraison, and the phospholipid hydroperoxide glutathione peroxidase enzyme may play a role in regulating the accumulation of (E)-2-hexenal [64]. In this study, the genotype effect plot for the markers surrounding the *PHOSPHOLIPID HYDROPEROXIDE GLUTATHIONE PEROXIDASE* gene indicated that the pollen grandparent ‘Seyval blanc’ contributed to the higher concentration of (E)-2-hexenal. It is possible that a higher activity or other differences in this phospholipid hydroperoxide glutathione peroxidase enzyme in *V. riparia* could limit the production of C_6_ aldehydes in contrast to the mechanism in ‘Seyval blanc’. However, further characterization of this enzyme in the grandparents, parent, and high and low concentration F_2_ is needed to determine whether phospholipid hydroperoxide glutathione peroxidase activity has a role in modulating the herbaceous volatile in the VRS-F_2_ population.

## 4. Materials and Methods

### 4.1. Plant Materials

Phenotypic data which comprise berry anthocyanin (2013 and 2018), malic acid and TA (2013, 2016, and 2018), and volatiles (2013 and 2018) were collected at 30 days post-veraison using three generations including grandparents, the F_1_ parent, and the VRS-F_2_ mapping population. The diploid VRS-F_2_ population was produced by selfing a single F_1_ (16_9_2) developed from a cross between *V. riparia* (seed parent, ‘Manitoba 37’, PI# 588289) and ‘Seyval blanc’ (pollen parent, VIVC#11558) [65]. The parent of the population (16_9_2) is a hermaphroditic genotype (having perfect flowers) with black berries, the grandparent *V. riparia* ‘Manitoba 37’ (USDA PI588259) is pistillate and produces black fruit while ‘Seyval blanc’, the pollen grandparent, is a hermaphroditic white-fruited wine grape. The initial 113 VRS-F_2_ progeny that were used to develop a previously reported SSR map are noted as field vines [65]. These 113 VRS-F_2_ vines, the parent, and pistillate grandparent, *V. riparia* ‘Manitoba 37’, were clonally propagated and planted in the vineyard at the N. E. Hansen Research Center, Brookings, SD (44.31° N, 96.80° W). Soils at the site were clay loam with 2% slopes. The vines were established in 2008 and spaced at 1.8 m apart in rows that were 3 m apart. Rows were oriented East–West with 48 vines per row. Weed, disease, and pest control were managed according to South Dakota industry standards. Due to low pressure, no fungicide or insecticide applications were conducted during the experimental period. Weed-free strips (0.6 m wide) were maintained below the vine rows with pre-emerge (Flumioxazin, Chateau^®^, Valent USA, San Ramon, CA, USA) and post-emerge (Glufosinate, Rely^®^, BASF, Florham Park, NJ, USA) herbicide applications. Red fescue (*Festuca rubra*) and clover (*Trifolium repens)* were grown between rows as a ground cover. Annual petiole tests were used in the vineyard to determine fertilizer applications.

### 4.2. VRS-F_2_ GBS-rhAmpSeq Integrated Genetic Map Construction

The VRS-F_2_ population was genotyped using the 2000 rhAmpSeq marker panel as described in Zou et al. 2020 [6], and 1970 pertinent rhAmpSeq markers were used with 1449 GBS markers Yang et al. 2016 [43] for this VRS-F_2_ GBS-rhAmpSeq-integrated map construction. The genotype frequencies of each marker were plotted (ggplot in R) against position on the genome for all chromosomes to identify regions of segregation distortion. The deviation from the expected Mendelian ratio (1:2:1) was then estimated using chi-square test *p*-values to detect individual marker segregation distortion. An appropriate threshold *p*-value for this population was then determined by testing eight *p*-values (0.05 (traditional *p*-value), 5 × 10^−3^, 1 × 10^−5^, 1 × 10^−10^, 1 × 10^−15^, 1 × 10^−21^, 1 × 10^−25^, 1 × 10^−30^) and visualizing marker loss relative to the adjusted *p*-value. JoinMap (version 5, Kyazma B. V., Wageningen, Netherlands) was used for GBS-rhAmpSeq integrated map construction [66]. After removing non-informative and distorted markers (chi-square adjusted *p*-value < 1 × 10^−21^), 1449 GBS and 1070 rhAmpSeq markers were used for map construction. Allele-calling errors were checked prior to map construction and suspect loci were manually corrected. A logarithm of odds (LOD) of five was used to establish linkage groups and Kosambi map function were used for map distance (in centimorgans, cM) calculations. Linkage group orientation was corrected using invert function if any inversions were found. Finally, the VRS-F_2_ GBS-rhAmpSeq linkage map was formatted into R/qtl ABH format in MS Excel, where ‘A’ and ‘B’ allele, respectively, represent major and minor homozygous alleles and ‘H’ is the heterozygous allele. To evaluate the VRS-F_2_ GBS-rhAmpSeq map, collinearity between the linkage map and the *V. vinifera* PN40024 12X V2 genome was measured by Spearman correlation coefficient (*cor.test* function in R) and visualized using correlation plot (*ggplot* function in R). A pair-wise recombination fraction heat map was generated using *plotRF* function (qtl package in R) to test marker order correctness. VRS-F_2_ GBS-rhAmpSeq linkage map imaging was performed using MapChart (2.32 version) [67].

### 4.3. Flower, Fruit, and Temperature Data 

Berry skin color was identified for 100 of the field sub-population vines. Berry quality data were collected using fruit harvested 30 days post-veraison in three different years. The berries were cut from the cluster leaving the peduncle attached. A random sample of 500 grams was collected from all harvested berries for each individual genotype, separated into two 250 g samples (one aliquot for TA, malic acid, and anthocyanins, and the other for volatiles) and stored at −20 °C until processing. Flower type was identified for 97 individuals from the field vines and used for QTL analysis. The markers identified in QTL were validated with a new set of flower type phenotypes collected in 2021 from 59 genotypes. The validated markers were used for the prediction of flower type in 358 additional genotypes with unknown flower type. Temperature data were collected from Brookings South Dakota Mesonet station for 2013, 2016 and 2018 growing seasons (June through August) covering flowering through harvest [68]. Growing degree days were calculated using hourly minimum and maximum temperatures and 50 °F base temperature.

### 4.4. Berry Titrable Acidity, Malic Acid, Anthocyanins, and Volatile Measurements

Replicate frozen berry samples (25 g) of frozen whole berries were destemmed and macerated for one min using a chilled 250 mL blender (Waring Laboratory Science, Stanford, CT, USA). Two 10 g portions transferred to 15 mL tubes and frozen at −20 °C for further use. TA at an endpoint of pH = 8.2 was determined by autotitrator (Titrino Plus 848 with a 869 autosampler, Metrohm, Riverview, FL, USA.). TA and malic acid were quantified for 2013, 2016 and 2018. Malic acid was quantified using a 10 g frozen berry macerate aliquot, which was thawed and centrifuged (5 min, 10,000× *g*) by a previously reported method. Briefly, a 10 g frozen berry macerate aliquot was thawed and centrifuged (5 min, 10,000× *g*). Juice samples were then injected onto an HPLC system (Agilent 1260; Santa Clara, CA, USA) consisting of a Bio-Rad micro-guard cation-H refill cartridge followed by a Bio-Rad Aminex HPX-87H ion exclusion column (Hercules, CA, USA). Malate was quantified by UV/VIS diode array detector at 210 nm. Calibrations were performed with malate acid standards, and repeatability (%RSD) was <3%. The 2013 malic acid phenotype was used previously in GBS map testing [43]; however, the 2013 malic acid results were incorporated in this manuscript to expand malic acid analyses by modeling the malic acid in three separate years (2013, 2016, and 2018). Anthocyanin extraction and quantitation for 2013 and 2018 samples were based on a method described by Manns and Mansfield [69]. A 10 g frozen berry macerate aliquot was thawed and centrifuged (5 min, 10,000× *g*), and the anthocyanin fraction isolated by solid-phase extraction (SPE) as described elsewhere [69]. HPLC analyses were performed on Agilent Model 1260 Infinity series on a Kinetex C18 column (100 mm × 4.6 mm, 2.6 μm particle size) fitted with a KrudKatcher guard filter (Phenomenex, Torrance, CA, USA). Mobile phase A was 0.5% *w/v* phosphoric acid in H_2_O, and mobile phase B was 0.5% phosphoric acid in methanol. The flow rate was 0.2 mL/min, and the gradient program was initially 15% B. Then, the flow rate increased linearly to 30% B at 15 min, then increased linearly to 60% B at 25 min, held at 60% B until 27 min, and then decreased linearly to 15% B at 30 min, after which the column was equilibrated for 10 min prior to the next injection. The column temperature was 45 °C. From the full UV/VIS spectrum (190–640 nm), absorbance data at 520 nm were used for anthocyanin quantitation. Anthocyanin identification was based on authentic standards for malvidin-3,5-diglucoside and malvidin-3-glucoside (Extrasynthese; Genay, France). For other anthocyanins (delphinidin-3,5-diglucoside, delphinidin-3-glucoside, cyanidin-3,5-diglucoside, cyanidin-3-glucoside, petunidin-3,5-diglucoside, petunidin-3-glucoside, peonidin-3,5-diglucoside, peonidin-3-glucoside), tentative identification was based on comparison of retention times to previously analyzed samples on the same instrument and using the same method, as described in an earlier report [69]. Previous work had established that this method achieved baseline resolution (R_f_ > 1.5) for all anthocyanins except for delphinidin-3-glucoside and petunidin-3,5-diglucoside (R_f_ ~0.8), with no evidence of other interferences in real samples. The method repeatability was evaluated by running malvidin-3-glucoside and malvidin-3,5-diglucoside standards at the start of run sets, and typical precisions were <1% RSD.

Quantification of herbaceous volatiles (hexanal, (E)-2-hexenal, and 3-isopropyl-2-methoxypyrazine (IPMP), in 2013 and 2018 berry samples was carried out by headspace solid phase microextraction (HS-SPME; LEAP CombiPALAutosampler Carrboro, NC. USA) coupled to a Shimadzu gas chromatograph-mass spectrometer (GC-MS) (GC2010 Plus w/TQ8040 MS; Nakagyo-ku, Kyoto, Japan) by adapting a method described by Burzynski-Chang et al. 2018 [70]. Frozen whole berries (25–50 g) were destemmed and macerated for one min using a chilled 250 mL stainless steel Waring blender. Berry macerates (5 g per vial, performed in duplicate) were transferred to 20 mL amber SPME vials (Sigma-Aldrich, St Louis, MO, USA) along with 3 g of NaCl, 5 mL 0.1 M pH 7.0 phosphate buffer, and 50 µL internal standard cocktail (initial concentrations prior to dilution = 118 mg/L d12-hexanal (CDN Isotopes, >98% purity; 99.1% isotopic purity), 8 mg/L d2-(E)-2-hexenal (Sigma-Aldrich, St. Louis, MO, USA), >90% purity; >99% isotopic purity), 9 µg/L d3-IPMP (aromaLAB), >98% purity; >99% isotopic purity). Identification of native compounds was performed with commercially purchased hexanal (Sigma-Aldrich, ≥97% purity), (E)-2-hexenal (Sigma-Aldrich, ≥95% purity), and 2-isopropyl-3-methoxypyrazine (IPMP) (Sigma-Aldrich, 97% purity). HS SPME analyses were performed using a 1 cm, 50/30 μm divinylbenzene-carboxen-polydimethylsiloxane (DVB/CARB/PDMS; Supleco, Bellafonte, PA, USA) with a pre-extraction incubation temperature of 60 °C for 15 min followed by a 15 min HS-SPME extraction. The SPME fiber was desorbed for 3 min in a split/splitless injector in splitless mode at a constant temperature of 230 °C, a purge time of 3 min and a purge flow of 50 mL/min. The GC column was a Varian Factor Four VF-WAXms (30 m × 0.25 mm × 0.25 µm) (Varian, Palo Alto, CA, USA) with helium as a carrier gas at a flow rate of 0.76 mL/min. The GC oven was held for 5 min at 40 °C, then ramped to 195 °C at 5 °C/min, then ramped to 240 °C at 20 °C/min and held for 5 min. The MS was operated in EI mode with an ionization energy of 70 eV. MS data were collected from *m/z* 25–250. Data processing was performed using Shimadzu GCMS Solutions Post-Run Analysis software. Concentrations of each volatile were calculated by determining the peak area ratio of the native analyte to its respective deuterated standard, and assuming that the response factor for the native compound was identical to its deuterated analogue. The quantifier ions as follows: for (E)-2-hexenal, *m/z* 83; for d2-(E)-2-hexenal, *m/z* 85; hexanal, *m/z* 72; for d12-Hexanal, *m/z* 79; for IPMP, *m/z* 152; and for d3-IPMP, *m/z* 155. To confirm selectivity, the ratios of the quantifier ion to the next two major ions (qualifier ions) were compared against the ratios for the authentic standards. To evaluate repeatability, selected samples (*n* = 5) were run in duplicate, and %RSD values < 30% were observed.

### 4.5. Statistical Data Analysis

Trait descriptive analysis was performed using psych [71] library in R statistical software [72]. Trait correlation analysis (Pearson) was conducted for berry acid traits using the stats library in R. Statistical significance was determined at *p*-value < 0.05 for descriptive and correlation analysis.

QTL mapping was performed using the VRS-F_2_ GBS-rhAmpSeq genetic map with 2519 markers across 19 chromosomes using R/qtl [73]. First, map validation was conducted using flower type and berry color phenotypes with binary QTL mapping method. QTL analyses were performed for malic acid, TA, total anthocyanin, total monoglucoside, total diglucoside, individual anthocyanins, and hexanal and (E)-2-hexenal volatiles. The normal model was used for quantitative traits meeting normality assumptions (as is or after transformation); however, if transformation of data did not meet normality assumptions, non-parametric QTL analysis was conducted. Normality evaluations and data transformation were performed using mass [74], gvlma [75] packages in R. Interval mapping was performed using the scanone function for each trait separately, using R/qtl package with three marker covariates, Kosambi mapping function, Haley-Knott regression and permutation test (1000, at alpha 0.1 and 0.05) to determine significant genome wide LOD thresholds for each trait analyzed. The confidence interval for each QTL was calculated using Bayesian method in R/qtl package at 95% confidence interval. The variation explained by each QTL was determined for each QTL using fitqtl function in R/qtl. Malic acid models were built for each year using all malic acid QTL identified, as well as testing peaks with LOD >3 using makeqtl function in R/qtl. QTL interactions were tested in modeling and significant interactions at 0.05 alpha were added to the model. Candidate gene protein alignment was conducted with Clustal Omega Multiple sequence alignment [76].

## 5. Conclusions

This study constructed a high-density, integrated VRS-F_2_ GBS-rhAmpSeq genetic map with 2519 markers across the 19 grapevine chromosomes. Natural segregation distortion was identified on chromosomes 5, 7, 11, and 15 during map development and marker testing. The inclusion of many markers that were identified as distorted at the standard 0.05 *p*-value provided a VRS-F_2_ GBS-rhAmpSeq map that was truer to the genetic structure of the inbred population. The greater marker density and rhAmpSeq markers provided greater opportunity to map traits and make comparisons with other interspecific populations. The map presented here identified remarkably narrow confidence intervals and candidate for qualitative traits, such as berry skin color (6.5 Mb) and flower type (0.2 Mb). Two transferable marker pairs ((one pistillate (rh_2_4497054 or rh_2_4599939) and one hermaphroditic (rh_2_4703733 or rh_2_4825658)) were identified to predict flower type in VRS-F_2_. Total anthocyanin and monoglucoside QTL occur on chromosome 2, with the monoglucoside peak marker located upstream of the berry color and total anthocyanins QTL peak positions. Total and individual diglucosides QTL were located on chromosome 9 with *5-O-GLUCOSYLTRANSFERASE* candidate gene (Vitvi09g00582) in repeat years. Our findings confirmed the presence of multiple acidity-related (malic acid and TA) QTL in one growing season. The malic acid QTL on chromosome 1 in 2016 and 2018 identified two additional malic acid related candidate genes (*MALATE DEHYDROGENASE*, Vitvi01g01744, Vitvi01g02239, Vitvi01g02240). The three seasons also confirmed stable QTL on chromosome 6, and ALMT (Vitvi06g00922, Vitvi06g00928) was the candidate gene in this interval. Modeling the QTL indicated multiple malic acid related genes on chromosome 1, 6, and 8 and explained >50% of the variation in 2016 and 2018. This study provides the first report of a volatile QTL and candidate gene *PHOSPHOLIPID HYDROPEROXIDE GLUTATHIONE PEROXIDASE* for (E)-2-hexenal, a grassy-smelling volatile that can have a negative herbaceous aroma at high concentrations. 

## Figures and Tables

**Table 1:** Integrated VRS-F_2_ GBS-rhAmpSeq map statistics.

Parameter	Value
Number of F_2_ genotypes	514
Total (GBS and rhAmpSeq) markers used in map curation	3428
Markers in LGs	2519
GBS markers in LGs	1449
rhAmpSeq markers in LGs	1070
LGs	19
Distortion threshold	1 × 10^−21^
Number of markers that formed different LG	0
Mismapped markers in LG	0
Markers not in any LG	0
Problematic markers in LG	0
Non-informative marker	677
Distorted marker numbers	232
Genetic map size (cM)	1131.3
Genome-wide recombination rate (cM/Mb)	2.5
Average distance between markers (cM)	0.5
Genome coverage (%)	96.3
Largest gap (cM)	11.3

LG, linkage group; cM, centimorgan; Mb, mega base pairs; %, percentage genome coverage, % coverage relative to *V. vinifera* ‘PN40024’ 12X V2 genome.

**Table 2:** Chromosome summary of the VRS-F_2_ genetic map.

Chromosome	Number of Markers	Chromosome Genetic Length (cM)	Average Spacing (cM)	Maximum spacing (cM)	Correlation (Spearman)
1	150	65.3	0.4	4.7	0.9999 *
2	112	52.2	0.5	4.2	0.9998 *
3	97	48.2	0.5	5.3	0.9996 *
4	181	62.7	0.3	2.3	0.9999 *
5	193	62.5	0.3	2.0	0.9999 *
6	131	60.1	0.5	4.9	0.9998 *
7	176	79.1	0.5	8.1	0.9999 *
8	145	59.3	0.4	2.8	0.9994 *
9	117	60.0	0.5	4.2	0.9999 *
10	88	55.9	0.6	11.3	0.9997 *
11	105	63.9	0.6	4.4	0.9996 *
12	124	56.5	0.5	3.3	0.9999 *
13	153	68.7	0.5	4.9	0.9999 *
14	161	68.9	0.4	3.1	0.9999 *
15	34	18.9	0.6	2.1	0.9997 *
16	114	55.5	0.5	3.1	0.9994 *
17	140	59.4	0.4	4.6	0.9998 *
18	192	79.9	0.4	8.5	0.9999 *
19	106	54.3	0.5	4.8	0.9999 *
Overall	2519	1131.3	0.5		

cM, centimorgan; Spearman, Spearman correlation coefficient; *, significant at *p*-value < 0.0001; Average spacing (cM) refers to the average genetic distance between two adjacent markers in each chromosome; Maximum spacing (cM) refers to the maximum genetic distance between two adjacent markers in each chromosome. Correlation refers to the relationship between genetic and physical length of the map.

**Table 3:** QTL for berry color, flower type, titratable acidity, malic acid, total anthocyanin, mono- and diglucoside anthocyanins and (E)-2-hexenal.

Trait	Chromosome	LOD	Peak Position (Physical Position (Mb))	R^2^	Physical (Mb) Position at 95% Bayesian Interval
Berry color	2	22.8	14.11	NP	11.00:17.48
Flower type	2	17.2	4.65	NP	4.50:4.70
Total anthocyanin 2013	2	9.4	13.54	NP	6.97:17.85
Total anthocyanin 2018	2	10.2	13.54	NP	6.97:17.85
Total anthocyanin 2013	18	3.4	6.94	NP	6.23:10.21
Total monoglucosides 2013	2	12.2	9.13	NP	8.58:14.87
Total monoglucosides 2018	2	3.8	5.87	NP	2.99:16:74
Total diglucosides 2013	2	5.7	8.11	NP	7.41:9.07
Total diglucosides 2013	9	4.8	6.19	NP	0.89:6.99
Total diglucosides 2018	9	3.2	6.52	NP	3.74:9.57
Malic acid 2013	6	4.5	11.92	28.2	2.31:15.48
Malic acid 2016	6	4.1	7.86	23.8	2.55:18.52
Malic acid 2018 ^a^	6	3.8	5.59	22.9	0.91:8.28
Malic acid 2018	1	5.2	20.62	31.0	18.88:23.67
Titratable acidity 2016 ^a^	1	3.8	6.29	22.5	0.97:8.38
Titratable acidity 2018	1	4.5	19.66	29.1	7.24:23.7
Titratable acidity 2013	6	4.8	15.25	29.7	2.85:16.63
Titratable acidity 2016	6	4.2	7.86	24.4	2.76:18.14
Titratable acidity 2018	6	4.1	5.59	28.1	0.28:18.52
(E)-2-hexenal 2013	2	4.4	7.47	29.4	5.35:18.70
(E)-2-hexenal 2018 ^a^	2	4.0	4.83	27.8	0.18:17.48

^a^ indicates significant at alpha test of 0.1 threshold, all others are significant at alpha test of 0.05 threshold; R^2^, percentage variation explained by the QTL; NP, parametric test was not conducted for this trait. Physical position Mb relative to PN40024 12X V2 genome.

## Data Availability

Data supporting the findings of this work are available within the paper and its Supplementary Information files. All the raw marker sequencing reads that support the findings of this study and its Supplementary Information file have been deposited in the in the National Center for Biotechnology Information Sequence Read Archive (SRA) and are accessible through BioProject ID PRJNA281110 [https://www.ncbi.nlm.nih.gov/bioproject/?term=PRJNA281110] (accessed on 28 December 2021).

## Supplementary Materials

The following are available online at https://www.mdpi.com/article/10.3390/plants11050696/s1 Table S1. VRS-F_2_ GBS-rhAmpseq genetic map coverage relative to *V. vinifera* PN40024 12X V2 reference genome. Table S2. Predicted pistillate (ff), homozygous hermaphrodite (HH) and heterozygous hermaphrodite (Hf) flower phenotype for VRS-F_2_ subset using linked markers. Table S3. Descriptive trait data for grandparents, parent, and VRS-F_2_ population. Table S4. Pearson correlation coefficient for malic acid and titratable acidity (TA) trait pairs. Table S5. Modeling of malic acid QTL increases explained variation for 2016 and 2018. Table S6. Growing degree days (GDD) from June through August in 2013, 2016, and 2018. Table S7. Individual mono- and diglucoside anthocyanins QTL Figure S1. Genotype frequency plots for all markers in 19 chromosomes. Dotted and dotdash lines represent the projected genotype count 128 (for AA and BB) and 256 (for AB) with an expected 1:2:1 Mendelian ratio. Figure S2. Number of genetic markers included with decreasing level of chi-square adjusted *p*-values. Figure S3. Collinearity between *V. vinifera* PN40024 V2 reference genome physical positions and genetic positions of the VRS-F_2_ GBS-rhAmpSeq linkage map. Figure S4. VRS-F_2_ GBS-rhAmpSeq genetic map. Chromosome number is at the top and black and red colors indicate GBS and rhAmpSeq markers, respectively (A, chromosome 1–5; B, chromosome 6–10; C, chromosome 11–15; D, Chromosome 16–19). Figure S5. Genotype accuracy relative to flower phenotype for a training set for potential flower type markers (n = 97).
