# Supplementary material for: Berry Anthocyanin, Acid, and Volatile Trait Analyses in a Grapevine-Interspecific F2 Population Using an Integrated GBS and rhAmpSeq Genetic Map
Source: Plants (Basel). 2022 Mar 4;11(5):696. doi: 10.3390/plants11050696 (PMC8912348; doi:10.3390/plants11050696)
Supplement: Supplementary file 1 [file plants-11-00696-s001.zip › plants-1553414-supplementary.pdf]

plants

MDPI

Table S1. VRS-F_2 GBS-rhAmpseq genetic map coverage relative to *V. vinifera* PN40024 12X V2 reference genome.

Chromosome	Start (bp)	end (bp)	Physical length (Mb)	PN40024 genome end (Mb)	Percent coverage compared to PN40024 genome
1	61203	23733091	23.67	24.23	97.7
2	99075	18702116	18.60	18.89	98.5
3	41401	19365074	19.32	20.70	93.4
4	130509	24606920	24.48	24.71	99.0
5	58210	25494850	25.44	25.65	99.2
6	226426	22260083	22.03	22.65	97.3
7	91133	27018992	26.93	27.36	98.4
8	30944	22458922	22.43	22.55	99.5
9	226145	22885244	22.66	23.01	98.5
10	34103	22647176	22.61	23.50	96.2
11	255751	20115217	19.86	20.12	98.7
12	173874	24257004	24.08	24.27	99.2
13	162233	29053865	28.89	29.08	99.4
14	88562	30108258	30.02	30.27	99.2
15	177997	12655885	12.48	20.30	61.5
16	3648	23550532	23.55	23.57	99.9
17	217227	18015932	17.80	18.69	95.2
18	227536	34381037	34.15	34.57	98.8
19	5269	24632229	24.63	24.70	99.7

bp, basepairs; Physical length (Mb) is chromosome length of 12X V2 *V. vinifera* 'PN40024' reference genome covered by this map. Coverage is calculated as the physical length presented for this linkage map divided by the total physical length of the chromosome in *V. vinifera* 'PN40024' 12X V2 genome.

Table S2. Predicted pistillate (ff), homozygous hermaphrodite (HH) and heterozygous hermaphrodite (Hf) flower phenotype for VRS-F_2 subset using linked markers.

Phenotype	rh_2_4497054	GBS_2_4567885	rh_2_4599939	GBS_2_4650201	rh_2_4703733	rh_2_4825658	GBS_2_5352479
Ff	91	89	89	88	90	92	90
HH	97	96	96	96	94	91	88
Hf	170	173	173	174	174	175	180

Table S3. Descriptive trait data for grandparents, parent, and VRS-F_2 population.

Trait	Year	Grandparent/ parent line			VRS-F_2 population		
		Vitis riparia 'Manitoba 37'	'Seyval blanc'	F_1 16_9_2	Mean	Min	Max
Total anthocyanin (mg/L)	2013	10799.8	0	264.0	3531.3	598.2	11186.8
	2018	Nd	nd	Nd	1020.6	49.6	2908.0
Total monoglucoside (mg/L)	2013	2624.8	0	964.9	1364.4	386.0	2886.2
	2018	Nd	nd	Nd	319.7	29.4	851.4
Total diglucoside (mg/L)	2013	8120.8	0	1658.4	2027.3	22.5	8344.1
	2018	Nd	nd	Nd	681.8	20.2	2187.8
Malic acid (g/L)	2013	14.7	nd	9.6	10.7	3.4	19.0
	2016	29.0	nd	12.6	12.0	4.1	23.4
	2018	12.8	nd	7.6	10.6	1.5	28.3
Titratable acidity (g/L)	2013	28.3	nd	21.1	22.5	14.8	35.8
	2016	26.0	nd	13.3	13.3	7.2	26.2
	2018	20.1	nd	14.8	12.9	6.0	24.0
(E)-2-hexenal (ppb)	2013	348.4	nd	225.8	406.8	60.5	1264.3
	2018	66.2	220.4	149.8	68.8	0.8	216.6
Hexanal (ppb)	2013	362.1	nd	205.0	318.3	82.8	825.5
	2018	1300.1	1370.5	714.8	925.6	354.6	2584.0
IPMP (ppb)	2013	21.6	nd	0	20.0	0.0	238.9
	2018	170.6	141.6	161.7	135.3	1.1	448.6
IBMP (ppb)	2013	39.6	nd	0	12.7	0.0	206.4

Sample size 2013 (63) 2016 (69), 2018 (64); Min, mini mum; Max, maximum; nd, data not available ('Seyval blanc' is subject to winter bud injury); mg, milligrams; L, liters; ppb, parts per billion

Table S4. Pearson correlation coefficient for malic acid and titratable acidity (TA) trait pairs.

Trait	TA 2013	TA 2016	TA 2018	Malic acid 2013	Malic acid 2016	Malic acid 2018
TA 2013	1.00	0.82*	0.82*	0.96*	0.84*	0.52*
TA 2016	0.82*	1.00	0.89*	0.78*	0.91*	0.62*
TA 2018	0.82*	0.89*	1.00	0.78*	0.90*	0.71*
Malic acid 2013	0.96*	0.78*	0.78*	1.00	0.83*	0.55*
Malic acid 2016	0.84*	0.91*	0.90*	0.83*	1.00	0.75*
Malic acid 2018	0.52*	0.62*	0.71*	0.55*	0.75*	1.00

*Significant at p-value < 0.0001

Table S5. Modeling of malic acid QTL increases explained variation for 2016 and 2018.

Trait	QTL model	Model LOD	R^2
Malic acid 2013	6@11920562	4.54	28.23
Malic acid 2016	1@6289520+6@7855019+8@10993976+1@6289520×8@10993976	13.66	59.83
Malic acid 2018	1@20356240+6@5589380+8@10343142	10.59	53.33

R^2, malic acid variation explained by the model; @, QTL peak position noted in bp relative to the *V. vinifera* 'PN40024' 12X V2 genome; × interaction.

Table S6. Growing degree days (GDD) from June through August in 2013, 2016, and 2018.

Year	Month	Monthly GDD	Cumulated GDD*
2013	June	457	457
	July	629	1087
	August	611	1698
2016	June	609	609
	July	646	1256
	August	606	1863
2018	June	616	616
	July	637	1253
	August	586	1840

*GDD calculated with 50 °F as base temperature.

Table S7. Individual anthocyanin QTL

Trait	Chromosome	LOD	Peak position (Mb)	Physical position (Mb) at the 95% Bayesian interval
Cyanidin 3-glucoside 2013	2	7.1	7.99	6.97:17.01
Malvidin 3-glucoside 2013	2	10.3	13.54	8.75:17.35
Malvidin 3-glucoside 2018	2	4.4	5.35	1.63:17.35
Peonidin 3-glucoside 2018	2	3.6	5.35	1.63:9.13
Petunidin 3-glucoside 2013	2	12.5	13.54	8.08:16.74
Petunidin 3-glucoside 2018	2	4.6	5.35	2.79:17.01
Cyanidin 3-glucoside 2018	9	3.5	6.52	3.74:9.57
Delphinidin 3-glucoside 2013	9	4.8	6.19	4.80:6.99
Peonidin 3-glucoside 2013	9	3.9	3.87	0.23:20.99
Malvidin 3-glucoside 2018	18	3.3	0.98	0.23:5.17
Delphinidin 3,5-diglucoside 2013	2	5.6	7.99	6.97:14.87
Malvidin 3,5-diglucoside 2013	2	7.1	7.99	6.97:17.01
Petunidin 3,5-diglucoside 2013	2	11.3	8.08	6.97:17.01
Cyanidin 3,5-diglucoside 2013	9	4.8	6.19	4.80:6.99
Cyanidin 3,5-diglucoside 2018	9	3.0	6.99	4.35:9.57
Delphinidin 3,5-diglucoside 2013	9	5.0	6.19	4.80:6.99
Malvidin 3,5-diglucoside 2013	9	3.3	6.19	0.23:6.99
Malvidin 3,5-diglucoside 2018	9	3.5	6.52	3.74:9.57
Peonidin 3,5-diglucoside 2013	9	4.8	6.19	4.80:6.99
Peonidin 3,5-diglucoside 2018	9	3.0	6.52	3.74:13.40
Petunidin 3,5-diglucoside 2013	9	3.4	6.52	0.89:7.34
Petunidin 3,5-diglucoside 2018	9	3.4	6.52	3.47:7.53
Cyanidin 3,5-diglucoside 2013	19	3.5	13.23	0.90:19.08

QTL significant at 1000 permutation alpha test of 0.05; Mb, physical position relative to *V. vinifera* 'PN40024' 12X V2 genome.

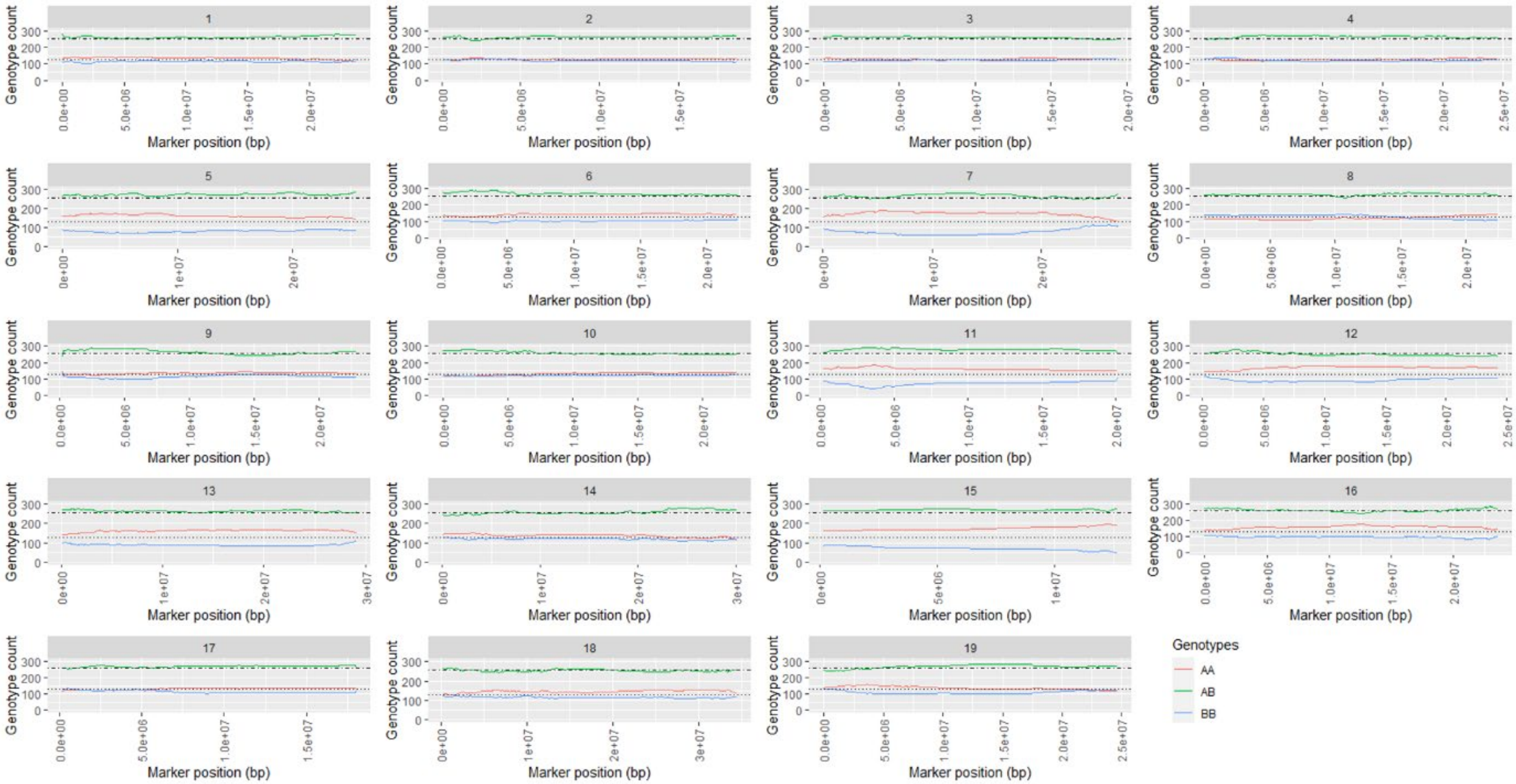

Figure S1. Genotype frequency plots for all markers in 19 chromosomes. Dotted and dotdash lines represent the expected genotype count 128 (for AA and BB) and 256 (for AB) under 1:2:1 Mendelian ratio

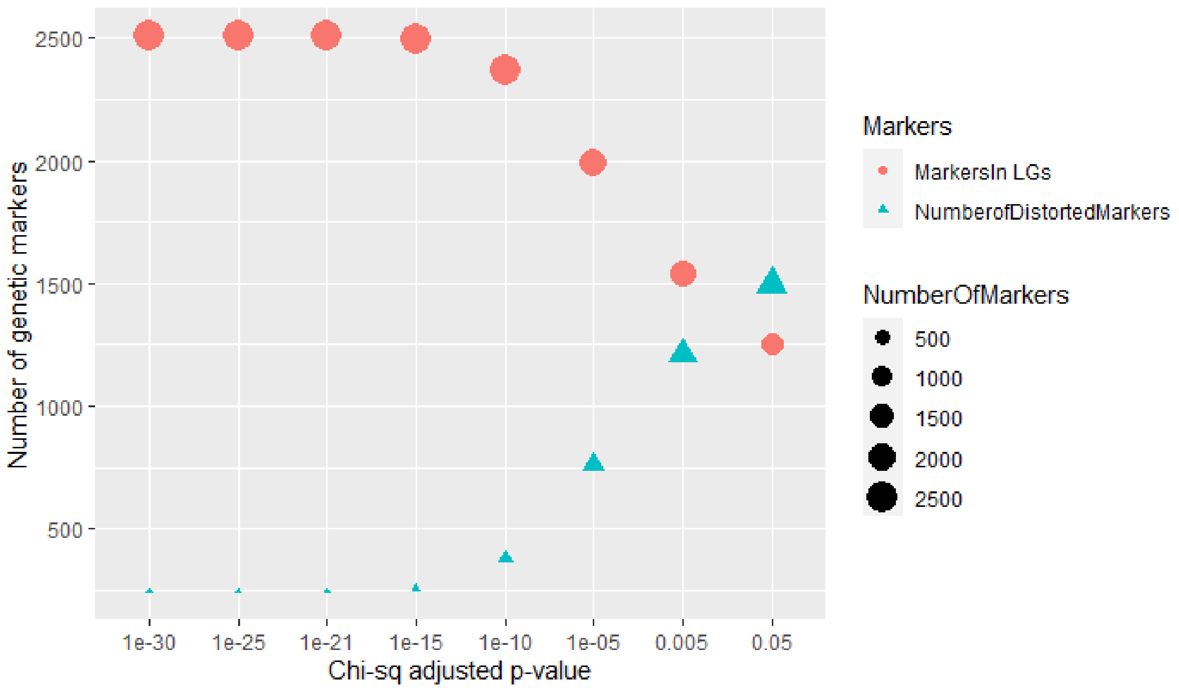

Figure S2. Number of genetic markers included with decreasing level of chi-square adjusted p-values.

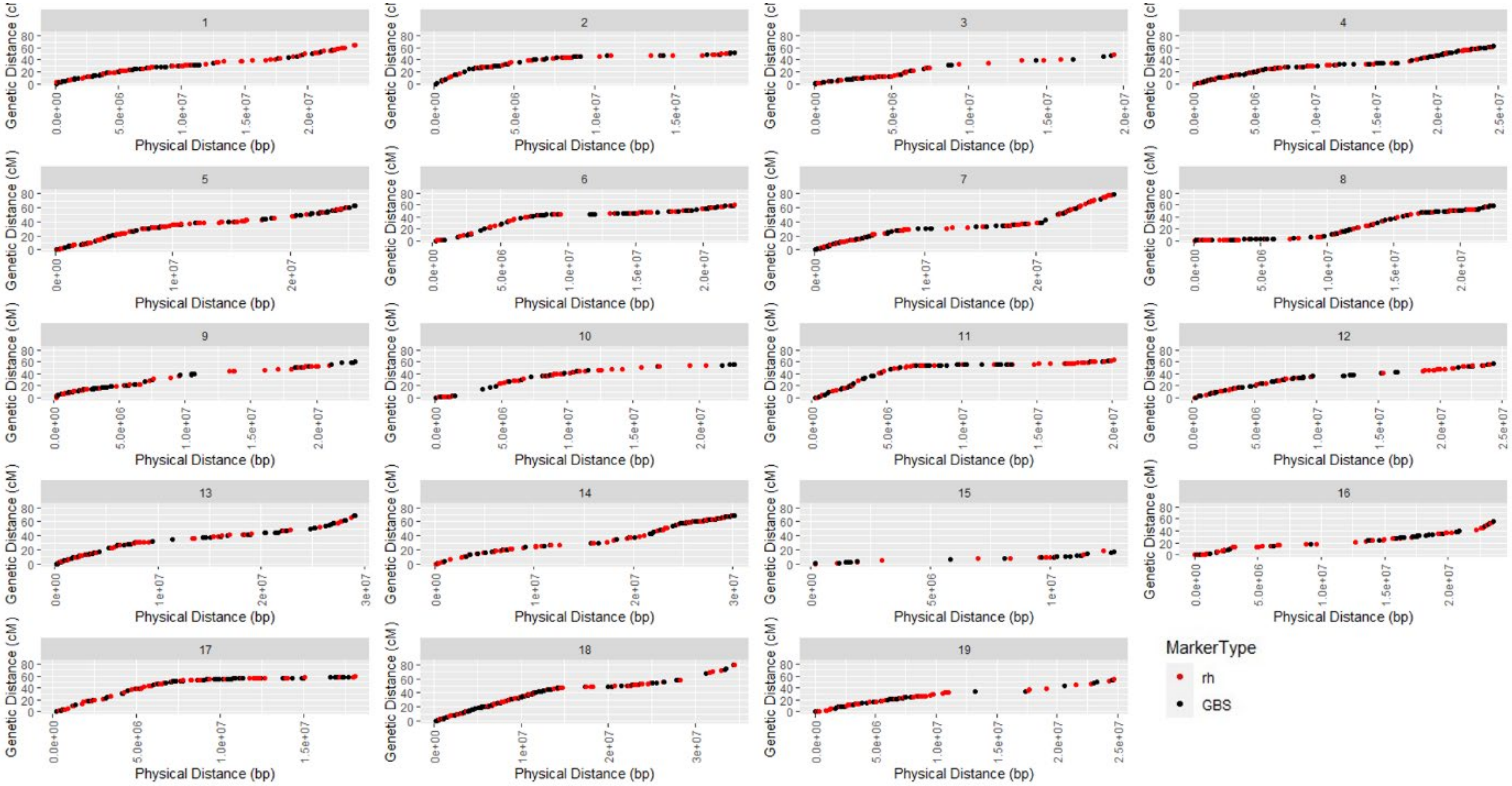

Figure S3. Collinearity between *V. vinifera* PN40024 V2 reference genome physical positions and genetic positions of the VRS-F_2 linkage map.

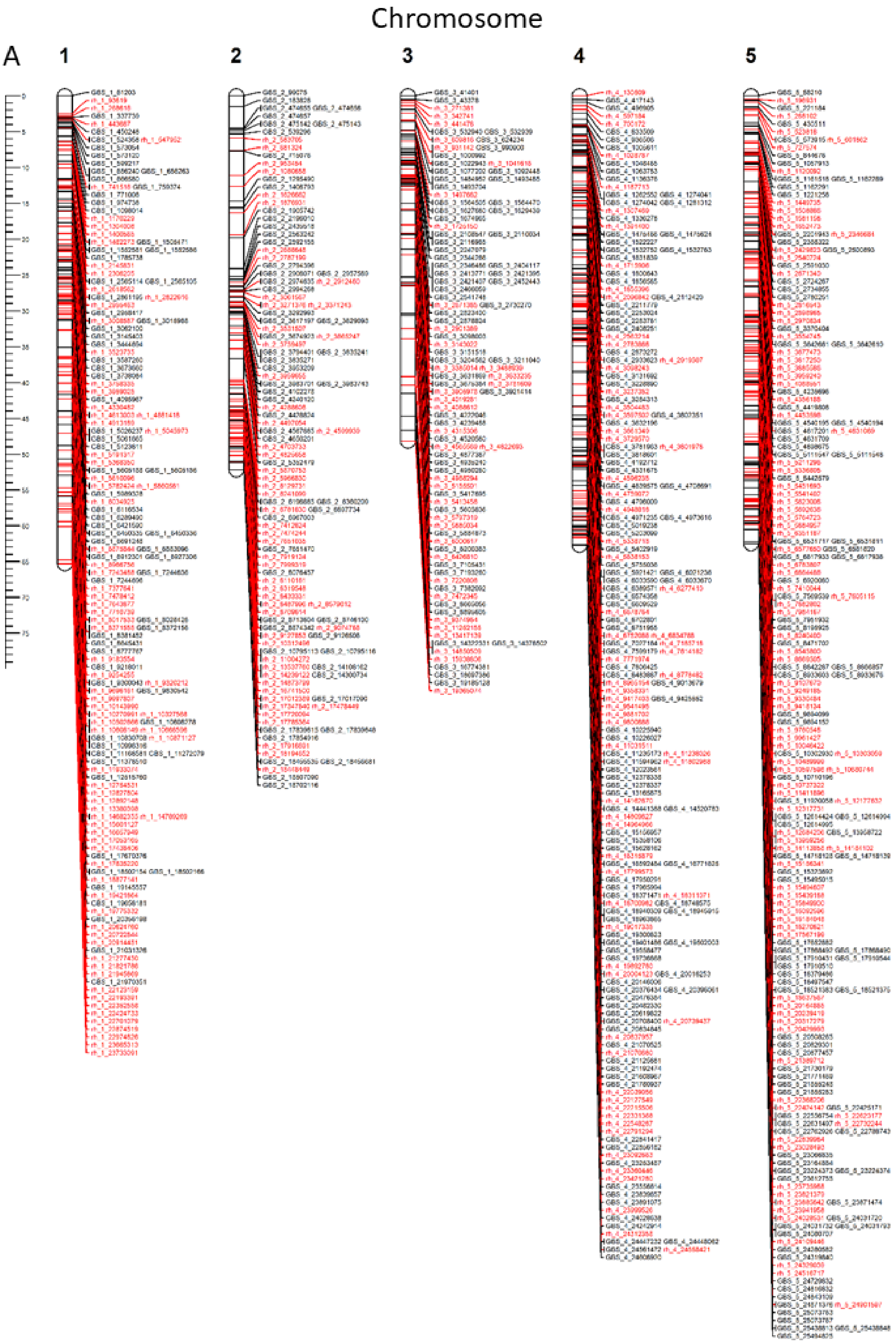
Chromosome
A
1
2
3
4
5

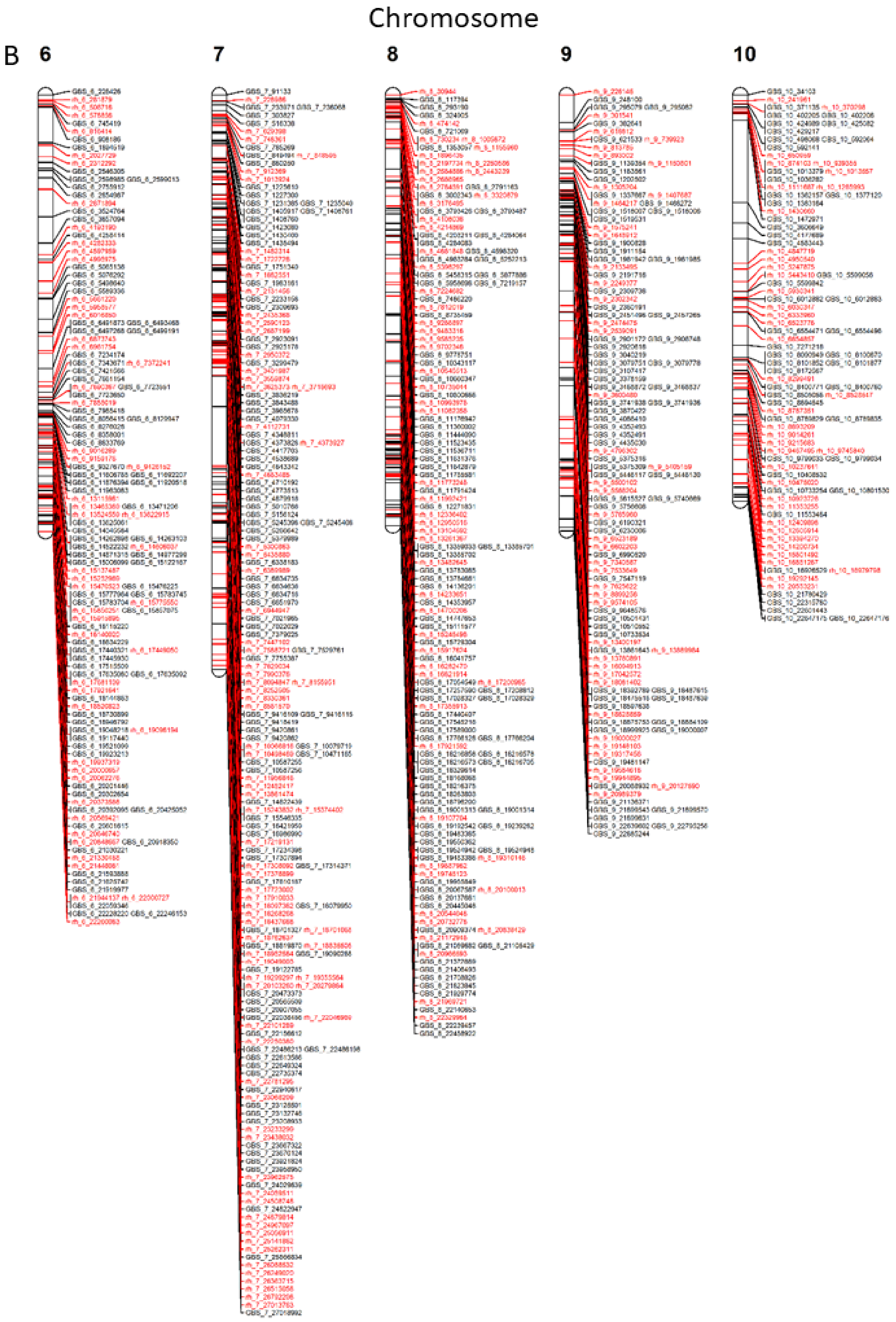
Chromosome
B
6
7
8
9
10

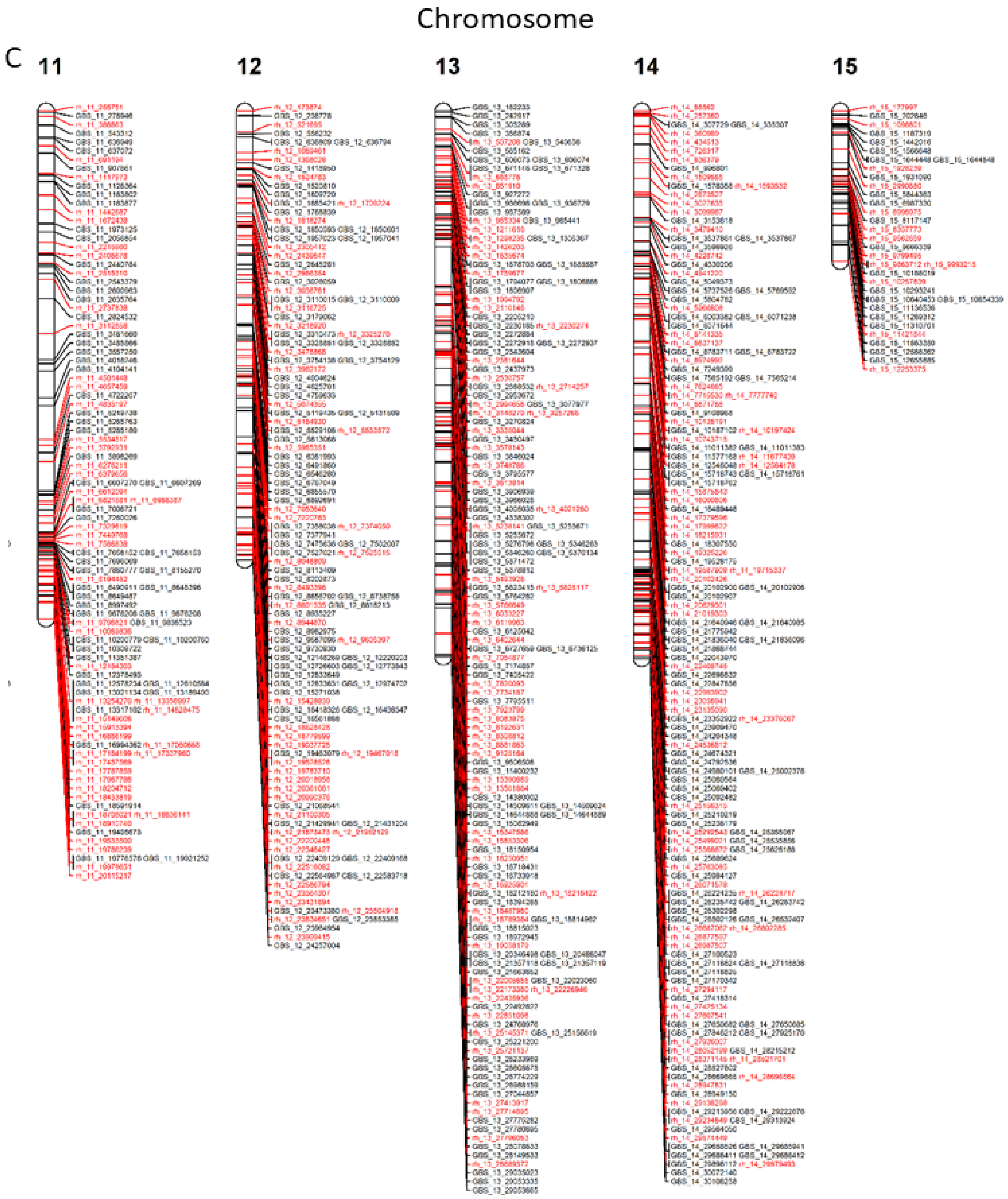
Chromosome
C
11
12
13
14
15

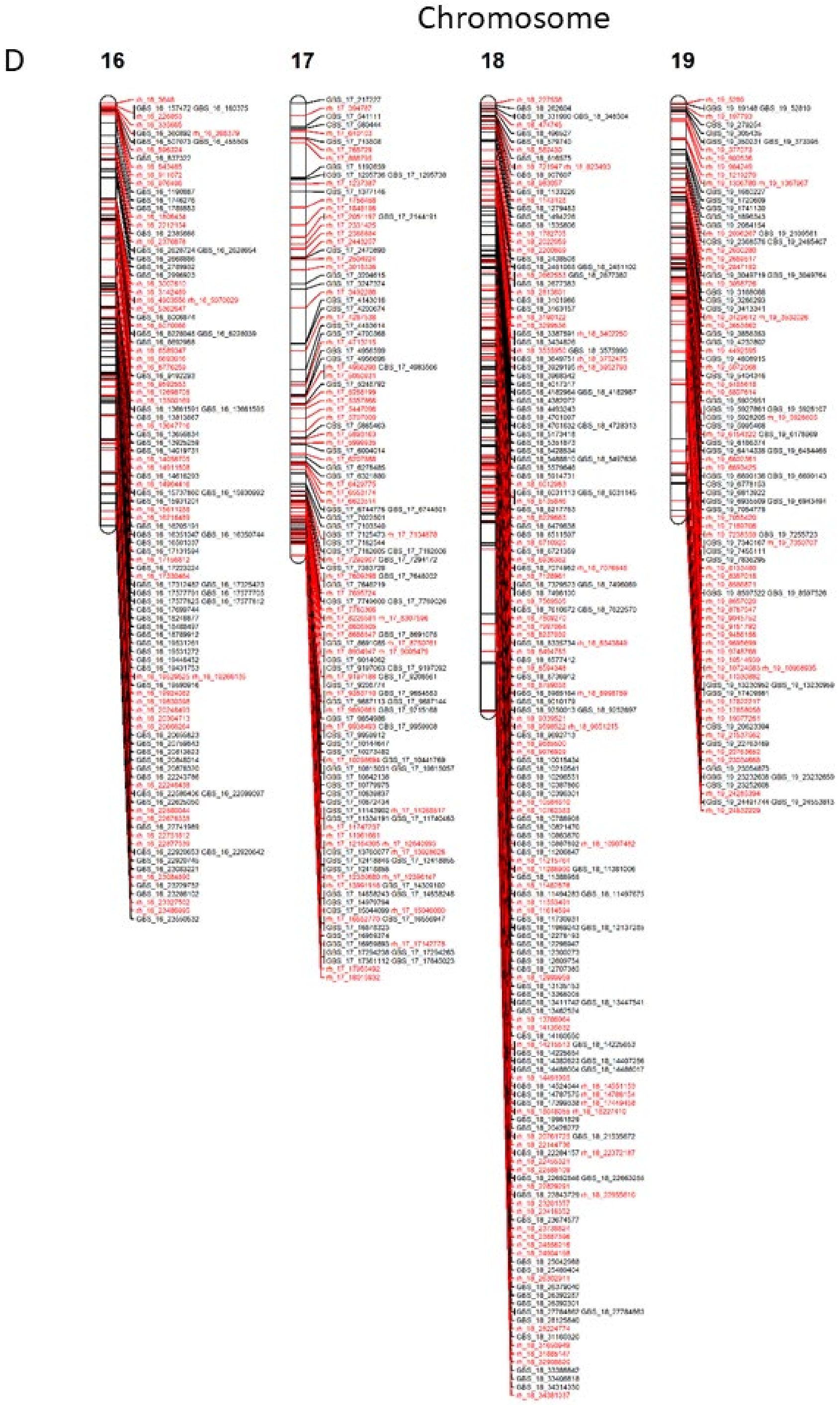

Figure S4. VRS-F2 GBS-rhAmpSeq genetic map. Chromosome number is at the top and black and red colors indicate GBS and rhAmpSeq markers, respectively.

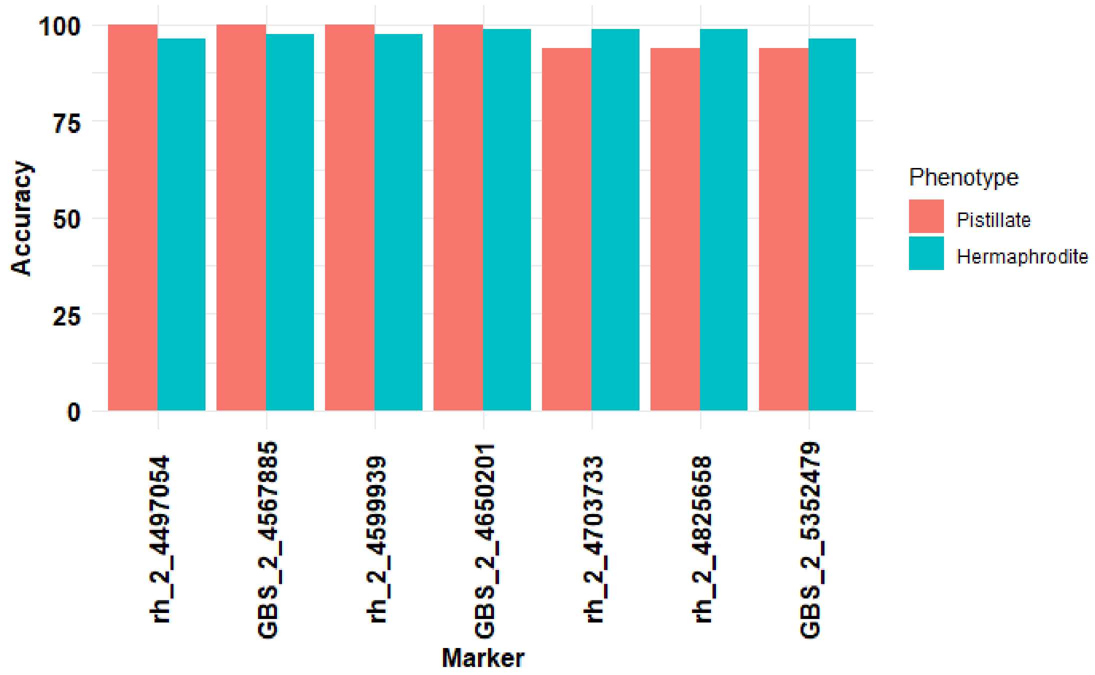

Figure S5. Genotype accuracy relative to flower phenotype in training set for potential flower type markers (n=97).

```
V.vinifera[Vitvi09g00582.t01]     MANPHPHFLIITFPAQGHINPALELAKRLIGVGADVTFATTIHAKSRLVKNPTVDGLRFS 60
V.riparia[XP_034695482.1]         MANPHPHFLIITFPAQGHINPALELAKRLIGVGADVTFATTIHAKSRLVKNPTVDGLRFS 60
V.amurensis[AHL68667.1]           MANPHPHFLIITFPAQGHINPALELAKRLIGVGADVTFATTIHAKSRLVKNPTVDGLRFS 60
V.rotundifolia[ALS55360.1]        MANPHPHFLIITFPAQGHIKPALELAKRLIGVGADVTFATTIHAKSRLVKNPTVDGLRFS 60
                                  *******************:****************************************

V.vinifera[Vitvi09g00582.t01]     TFSDGQEEGVKRGPNDLPVFQRLASENLSELIMASANEGRPISCLIYSIVIPGAAELARS 120
V.riparia[XP_034695482.1]         TFSDGQEEGVKRGPNDLPVFQRLASENLSELIMASANEGRPISCLIYSILIPGAAELARS 120
V.amurensis[AHL68667.1]           TFSDGQEEGVKRGPNELPVFQRLASENLSELIMASANEGRPISCLIYSILIPGAAELARS 120
V.rotundifolia[ALS55360.1]        TFSDGQEEGVKRGPNELPVFQRLASENLSELIMASANEGRPISCLIYSILIPGAAELARS 120
                                  ***************:*********************************:**********

V.vinifera[Vitvi09g00582.t01]     FNIPSAFLWIQPATVLDIYYYYFNGFGDLIRSKSSDPSFSIELPGLPSLSRQDLPSFFVG 180
V.riparia[XP_034695482.1]         FNIPSAFLWIQPATVLDIYYYYFNGFGDLIRSKSSDPSFSIELPGLPSLSRQGLPSFFVG 180
V.amurensis[AHL68667.1]           FNIPSAFLWIQPATVLDIYYYYFNGFGDLIRSKSSDPSFSIELPGLPSLSRQDLPSFFVG 180
V.rotundifolia[ALS55360.1]        FNIPSAFLWIQPATVLDIYYYYFNGFGDLIRSKSSDPSFSIELPGLPSLSRQDLPSFFVG 180
                                  ****************************************************.*******
V.vinifera[Vitvi09g00582.t01]     SDQNQENHALAAFQKHLEILEQEENPKALVNTFDALEPEALRAVEKLKLTAVGPLVPSGF 240
V.riparia[XP_034695482.1]         SDQNQENHALAAFQKHLEILEQEENPKVLVNTFDALEPEALRAVEKLKLTAVGPLVPSGF 240
V.amurensis[AHL68667.1]           SDQNQENHALAAFQKHLEILEQEENPKVLVNTFDALEPEALRAVEKLKLTAVGPLVPSGF 240
V.rotundifolia[ALS55360.1]        SDQNQENHALAAFQKHLEILEQEENPKVLVNTFDALEPEALRAVEKLKLTAVGPLVPSGF 240
                                  ***************************.********************************

V.vinifera[Vitvi09g00582.t01]     SDGKDASDTPSGGDLSDGSRDYMEWLKSKPESTVVYVSFGSISMFSMQQMEEIARGLLES 300
V.riparia[XP_034695482.1]         SDGKDASDTPSGGDLSDGSRDYMEWLKSKPESTVVYVSFGSISMFSMQQMEEIARGLLES 300
V.amurensis[AHL68667.1]           SDGKDASDTPSGGDLSDGSRDYMEWLKSKPESTVVYVSFGSISMFSMQQMEEIARGLLES 300
V.rotundifolia[ALS55360.1]        SDGKDASDTPSGGDLSDGSRDYMEWLKSKPESTVVYVSFGSISMFTMQQMEEIARGLLES 300
                                  *********************************************:**************

V.vinifera[Vitvi09g00582.t01]     GRPFLWLIRAKENGEENKEEDKLSCQEELEKQGMLIQWCSQMEVLSHPSLGCFVTHCGWN 360
V.riparia[XP_034695482.1]         GRPFLWVIRAKENGEENKEEDKLSCQEELEKQGMLIQWCSQMEVLSHPSLGCFVTHCGWN 360
V.amurensis[AHL68667.1]           GRPFLWVIRAKENGEENKEEDKLSCQEELEKQGMLIQWCSQMEVLSHPSLGCFVTHCGWN 360
V.rotundifolia[ALS55360.1]        GRPFLWVIRAKENGEENKEEDKLSCQEELEKQGMLIQWCSQMEVLSHPSLGCFVTHCGWN 360
                                  ******:*****************************************************

V.vinifera[Vitvi09g00582.t01]     SSIESLASGVPMIAFPQWADQGTNTKLIKDVWKTGVRLMVNEEEIVTSDELKR------- 413
V.riparia[XP_034695482.1]         SSIESLASGVPMIAFPQWADQGTNTKLIKDVWKTGVRLMVNEEEIVTSDELKRCLELVMG 420
V.amurensis[AHL68667.1]           SSIESLASGVPMIAFPQWADQGTNTKLIKDVWKTGVRLMVNEEEIVTSDELRRCLELVMG 420
V.rotundifolia[ALS55360.1]        SSIESLASGVPMIAFPQWADQGTNTKLIKDVWKTGVRLMVNEEEIVTSDELRRCLELVMG 420
                                  ***************************************************:*

V.vinifera[Vitvi09g00582.t01]     --------------------------------------------       413
V.riparia[XP_034695482.1]         DGEKGQEMRKNAKKWKILTKEALKEGGSSHKNLKNFVDEVIQGY       464
V.amurensis[AHL68667.1]           DGEKGQEMRKNAKKWKILAKEALKEGGSSHKNLKNFVDEVIQGY       464
V.rotundifolia[ALS55360.1]        DGEKGQEMRKNAKKWKILAKEALKEGGSSHKNLKNFVDEVILGY       464
```

Figure S6. Protein alignment of *V. vinifera, V. riparia, V. amurensis,* and *V. rotundifolia anthocyanin 5-O-glycosyltranserase*. Gray highlights a 51 amino acid truncation in *V. vinifera* PN40024 12X V2, Vitivi09g000582. NCBI Accession numbers are identified in brackets following species name.

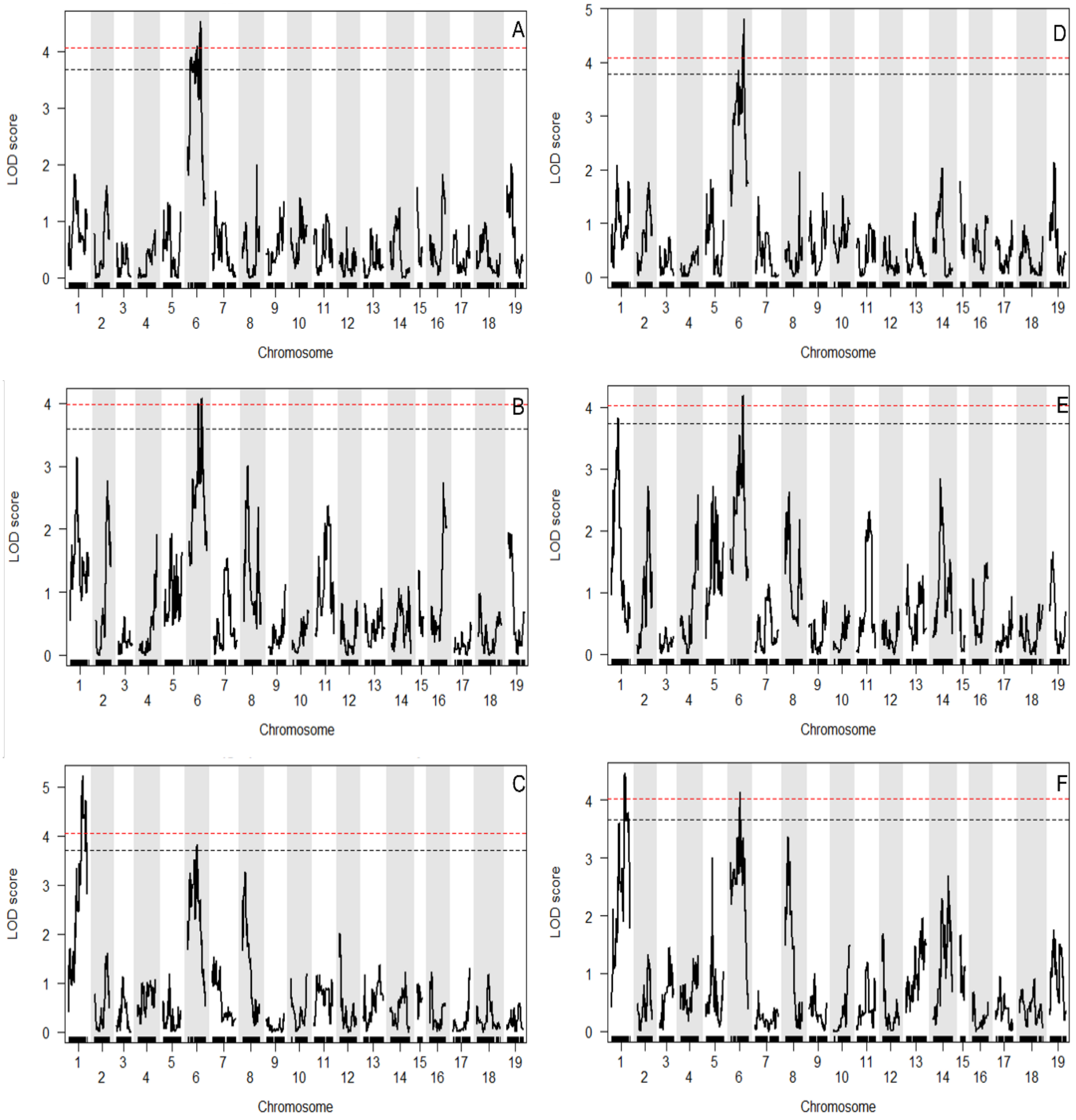

Figure S7. Genome-wide LOD score for malic acid (2013 (A), 2016 (B), and 2018 (C)) and titratable acidity (TA) (2013 (D), 2016 (E), and 2018 (F)). Black and red dashed horizontal lines represent 1000 permutation test at alpha of 0.1 and 0.05, respectively.
